# Confirming the attainment of maximal oxygen uptake within special and clinical groups: A systematic review and meta-analysis of cardiopulmonary exercise test and verification phase protocols

**DOI:** 10.1371/journal.pone.0299563

**Published:** 2024-03-28

**Authors:** Victor A. B. Costa, Adrian W. Midgley, Julia K. Baumgart, Sean Carroll, Todd A. Astorino, Gustavo Z. Schaun, Guilherme F. Fonseca, Felipe A. Cunha

**Affiliations:** 1 Graduate Program in Exercise Science and Sports, University of Rio de Janeiro State, Rio de Janeiro, Brazil; 2 Laboratory of Physical Activity and Health Promotion, University of Rio de Janeiro State, Rio de Janeiro, Brazil; 3 Department of Sport and Physical Activity, Edge Hill University, Ormskirk, England, United Kingdom; 4 Centre for Elite Sports Research, Department of Neuromedicine and Movement Science, Norway, University of Science and Technology, Trondheim, Norway; 5 School of Sport, Exercise and Rehabilitation Sciences, University of Hull, Hull, England, United Kingdom; 6 Department of Kinesiology, California State University, San Marcos, CA, United States of America; 7 Centre for Sport Science and University Sports, University of Vienna, Vienna, Austria; Hamad Bin Khalifa University, QATAR

## Abstract

**Background and aim:**

A plateau in oxygen uptake ($\dot{V}O_{2}$) during an incremental cardiopulmonary exercise test (CPET) to volitional exhaustion appears less likely to occur in special and clinical populations. Secondary maximal oxygen uptake ($\dot{V}O_{2\max}$) criteria have been shown to commonly underestimate the actual $\dot{V}O_{2\max}$. The verification phase protocol might determine the occurrence of ‘true’ $\dot{V}O_{2\max}$ in these populations. The primary aim of the current study was to systematically review and provide a meta-analysis on the suitability of the verification phase for confirming ‘true’ $\dot{V}O_{2\max}$ in special and clinical groups. Secondary aims were to explore the applicability of the verification phase according to specific participant characteristics and investigate which test protocols and procedures minimise the differences between the highest $\dot{V}O_{2}$ values attained in the CPET and verification phase.

**Methods:**

Electronic databases (PubMed, Web of Science, SPORTDiscus, Scopus, and EMBASE) were searched using specific search strategies and relevant data were extracted from primary studies. Studies meeting inclusion criteria were systematically reviewed. Meta-analysis techniques were applied to quantify weighted mean differences (standard deviations) in peak $\dot{V}O_{2}$ from a CPET and a verification phase within study groups using random-effects models. Subgroup analyses investigated the differences in $\dot{V}O_{2\max}$ according to individual characteristics and test protocols. The methodological quality of the included primary studies was assessed using a modified Downs and Black checklist to obtain a level of evidence. Participant-level $\dot{V}O_{2}$ data were analysed according to the threshold criteria reported by the studies or the inherent measurement error of the metabolic analysers and displayed as Bland-Altman plots.

**Results:**

Forty-three studies were included in the systematic review, whilst 30 presented quantitative information for meta-analysis. Within the 30 studies, the highest mean $\dot{V}O_{2}$ values attained in the CPET and verification phase protocols were similar (mean difference = -0.00 [95% confidence intervals, CI = -0.03 to 0.03] L·min^-1^, *p* = 0.87; level of evidence, LoE: strong). The specific clinical groups with sufficient primary studies to be meta-analysed showed a similar $\dot{V}O_{2\max}$ between the CPET and verification phase (*p* > 0.05, LoE: limited to strong). Across all 30 studies, $\dot{V}O_{2\max}$ was not affected by differences in test protocols (*p* > 0.05; LoE: moderate to strong). Only 23 (53.5%) of the 43 reviewed studies reported how many participants achieved a lower, equal, or higher $\dot{V}O_{2}$ value in the verification phase versus the CPET or reported or supplied participant-level $\dot{V}O_{2}$ data for this information to be obtained. The percentage of participants that achieved a lower, equal, or higher $\dot{V}O_{2}$ value in the verification phase was highly variable across studies (e.g. the percentage that achieved a higher $\dot{V}O_{2}$ in the verification phase ranged from 0% to 88.9%).

**Conclusion:**

Group-level verification phase data appear useful for confirming a specific CPET protocol likely elicited $\dot{V}O_{2\max}$, or a reproducible $\dot{V}O_{2peak}$, for a given special or clinical group. Participant-level data might be useful for confirming whether specific participants have likely elicited $\dot{V}O_{2\max}$, or a reproducible $\dot{V}O_{2peak}$, however, more research reporting participant-level data is required before evidence-based guidelines can be given.

**Trial registration:**

PROSPERO (CRD42021247658) https://www.crd.york.ac.uk/prospero.

## Introduction

Maximal oxygen uptake ($\dot{V}O_{2\max}$) represents the upper physiological limit of utilising oxygen to produce energy during volitional exercise to exhaustion [1]. The original concept emerged in the 1920’s in the seminal works of Hill and colleagues [2, 3]. These authors described this phenomenon as the “ceiling” of oxygen uptake ($\dot{V}O_{2}$) during a discontinuous step-incremented exercise test, beyond which no additional increase in $\dot{V}O_{2}$ is observed despite an increase in work rate. In special groups such as apparently healthy children and older adults, and clinical groups such as people with chronic respiratory and metabolic conditions, $\dot{V}O_{2\max}$ testing is increasingly recommended and typically determined using an incremental cardiopulmonary exercise test (CPET) with concurrent recording of electrocardiography, blood pressure, and oxyhemoglobin saturation [4]. Applications of CPET include facilitating the diagnosis and evaluating the physiological impact of cardiopulmonary disease [5], evaluating fitness for major surgery [4], deciding on the appropriateness of cardiac transplantation [6], investigating unexplained dyspnea and exercise intolerance [4], prescribing exercise to special and clinical groups [7, 8], and evaluating the chronic effects of exercise training programs and other interventions to promote health [9]. Over 40 outcome variables can be derived from a CPET [10], however, $\dot{V}O_{2\max}$ is widely regarded as the most important, since it is considered the gold-standard measure of cardiorespiratory fitness [4]. The importance of cardiorespiratory fitness for health and longevity is highlighted by the scientific statement from the American Heart Association, which calls for cardiorespiratory fitness to be recognised as a “clinical vital sign” [11].

Different ergometers, test protocols, procedures, and criteria have been developed and applied to promote accurate $\dot{V}O_{2\max}$ determination, which is significant considering the relationship between $\dot{V}O_{2\max}$ and health status. Potential reasons for not obtaining valid $\dot{V}O_{2\max}$ values include inappropriate test protocols and poor effort from participants [12]. However, there is no consensus on the best approach to establish whether a ‘true’ $\dot{V}O_{2\max}$ has been attained. The primary criterion for verifying that $\dot{V}O_{2\max}$ has been attained has been based on establishing a $\dot{V}O_{2}$ plateau at volitional exhaustion, first proposed in 1955 by Taylor, Buskirk and Henschel [13]. They defined the plateau occurrence as an increase in $\dot{V}O_{2}$ of less than 150 mL·min^-1^ (or ≤ 2.1 mL·kg^-1^·min^-1^) during a discontinuous step-incremented protocol incorporating 3–5 visits to the laboratory. With advances in technology (specifically, gas analysers that record breath-by-breath pulmonary gas exchange data and electronically programmable treadmills and cycle ergometers), discontinuous step-incremented tests protocols have largely been replaced by more time-efficient continuous ramp and pseudo-ramp protocols [14–17]. Ramp protocols typically elicit an accelerated or linear $\dot{V}O_{2}$ response during the final portion of the test, which reduces plateau incidence, especially in low fit individuals [18], although a high level of variability has been observed between studies [19–21]. This variability can be explained by the use of different $\dot{V}O_{2}$ plateau criterion thresholds [22], $\dot{V}O_{2}$ sampling intervals [23], test protocols [24], and variation in participant characteristics [25]. This inherent dependency on the test protocol and procedures, and participant characteristics, reduces the robustness of the $\dot{V}O_{2}$ plateau concept for confirming $\dot{V}O_{2\max}$ attainment.

Numerous investigators have not applied a $\dot{V}O_{2}$ plateau criterion or have relied upon so-called secondary criteria to assess whether $\dot{V}O_{2\max}$ has been elicited if a $\dot{V}O_{2}$ plateau threshold has not been met. Examples of secondary $\dot{V}O_{2\max}$ criteria include attainment of 90% age-predicted maximal heart rate, maximal respiratory exchange ratio ≥ 1.10, post-exercise blood lactate concentration ≥ 8 mmol·L^-1^, and ratings of perceived exertion ≥ 18 [12, 26]. However, secondary $\dot{V}O_{2\max}$ criteria may result in an underestimation of $\dot{V}O_{2\max}$, given several studies have observed that participants often satisfy the thresholds at submaximal exercise intensities [20, 22, 27]. Moreover, like the $\dot{V}O_{2}$ plateau, secondary $\dot{V}O_{2\max}$ criteria are dependent on the test protocol and procedures and participant characteristics [12]. Some researchers have therefore encouraged the abandonment of traditional primary and secondary $\dot{V}O_{2\max}$ criteria due to their lack of sensitivity and specificity in establishing whether a ‘true’ $\dot{V}O_{2\max}$ has been attained [20, 27].

The verification phase is an increasingly recognised procedure for confirming ‘true’ $\dot{V}O_{2\max}$ and typically involves performing a subsequent constant work rate (square wave), sub- or supra- peak bout of exercise after the CPET has been voluntarily terminated (incorporating a short or more extended rest period in between the tests) [20, 27]. The supra peak verification phase is conceptually like the discontinuous $\dot{V}O_{2\max}$ tests that were used in developing $\dot{V}O_{2}$ plateau criteria, but has the advantage of requiring only one visit to the laboratory [28]. The verification phase has emerged as a potentially valid alternative for establishing whether a ‘true’ $\dot{V}O_{2\max}$ has been attained [28, 29]. A recent meta-analysis of studies recruiting apparently healthy participants reported that unlike traditional $\dot{V}O_{2\max}$ criteria, the verification phase is not affected by the $\dot{V}O_{2\max}$ test protocol or procedures, or participant characteristics such as sex and level of cardiorespiratory fitness [29]. Considering the $\dot{V}O_{2}$ plateau is less likely to occur in unfit participants, and that secondary criteria commonly underestimate $\dot{V}O_{2\max}$ in special and clinical groups [30–32], the verification phase might be particularly useful to establish the occurrence of ‘true’ $\dot{V}O_{2\max}$ or, when $\dot{V}O_{2\max}$ has not been elicited, the highest possible attainable $\dot{V}O_{2}$ in these groups. Notably, participant and test protocol characteristics may not allow the attainment of $\dot{V}O_{2\max}$ during the CPET or verification phase. This has direct clinical applications in establishing functional capacity and the effectiveness of exercise training, and the subsequent evaluation of health risks in clinical populations. However, no systematic review and meta-analysis has investigated the utility of the verification phase across diverse special and clinical groups according to different CPET and verification phase protocols and procedures. The effect of these factors on the utility of the verification phase in special and clinical groups is therefore unclear.

The primary aim of the current study was to systematically review and provide a meta-analysis on the suitability of the verification phase for confirming ‘true’ $\dot{V}O_{2\max}$ in special and clinical groups. Secondary aims were to explore the applicability of the verification phase according to specific participant characteristics and investigate which test protocols and procedures minimise the differences between the highest $\dot{V}O_{2}$ values attained in the CPET and verification phase.

## Methods

### Protocol and registration

The systematic review and meta-analysis were performed and reported in accordance with the Preferred Reporting Items for Systematic Reviews and Meta-Analyses (PRISMA) [33]. A full PRISMA checklist is shown in S1 Checklist. The protocol was registered at https://www.crd.york.ac.uk/prospero (CRD42021247658).

### Search strategy

MEDLINE (accessed through PubMed), Web of Science, SPORTDiscus, Scopus, and EMBASE were searched for peer-reviewed literature. The search strategy included terms relating to cardiopulmonary exercise test, verification phase, $\dot{V}O_{2\max}$ test, and oxygen uptake, using a combination of entry terms and synonyms. Medical subject heading (MeSH) descriptors were also included in the PubMed search. All studies published from the inception of the databases until the search date (14^th^ October 2023) were sought. All references from electronic search results were imported into Endnote bibliographic software (version X9, Bld 12062, Clarivate) and duplicates were removed. The electronic searches were re-run before the final analysis and further studies were retrieved for inclusion. A list containing the full search strategy for each database is available (see S1 Fig). Backward searching for additional relevant studies was conducted by scrutinising the reference lists of the full-text articles of the initial included studies. Forward searching for additional relevant studies was conducted within electronic databases by scrutinising studies that have cited the initial included studies since their publication.

Eligibility criteria for inclusion were studies published in English or Portuguese language involving 1) *Population*–individuals affected by any disease, disability or clinical condition, apparently healthy children and adolescents (<18 years of age), and older adults (≥ 65 years of age) according to American College of Sports Medicine and American Heart Association definitions [34]; 2) *Type of study*–any research design that included at least one CPET and at least one verification phase carried out on a cycle ergometer, while walking or running or wheelchair propulsion on a treadmill, or on a ski-/wheelchair-/arm-ergometer; and 3) *Outcome*– $\dot{V}O_{2\max}$ determined using expired gas analysis during the maximal CPET (control) and verification phase (comparator). Studies were excluded if: 1) they involved secondary analysis of previously included studies; and 2) they investigated older adults, and it could not be ascertained whether any of the participants were below 65 years old. Two blinded researchers performed the searches and screening procedures.

### Study selection

**Fig 1:**
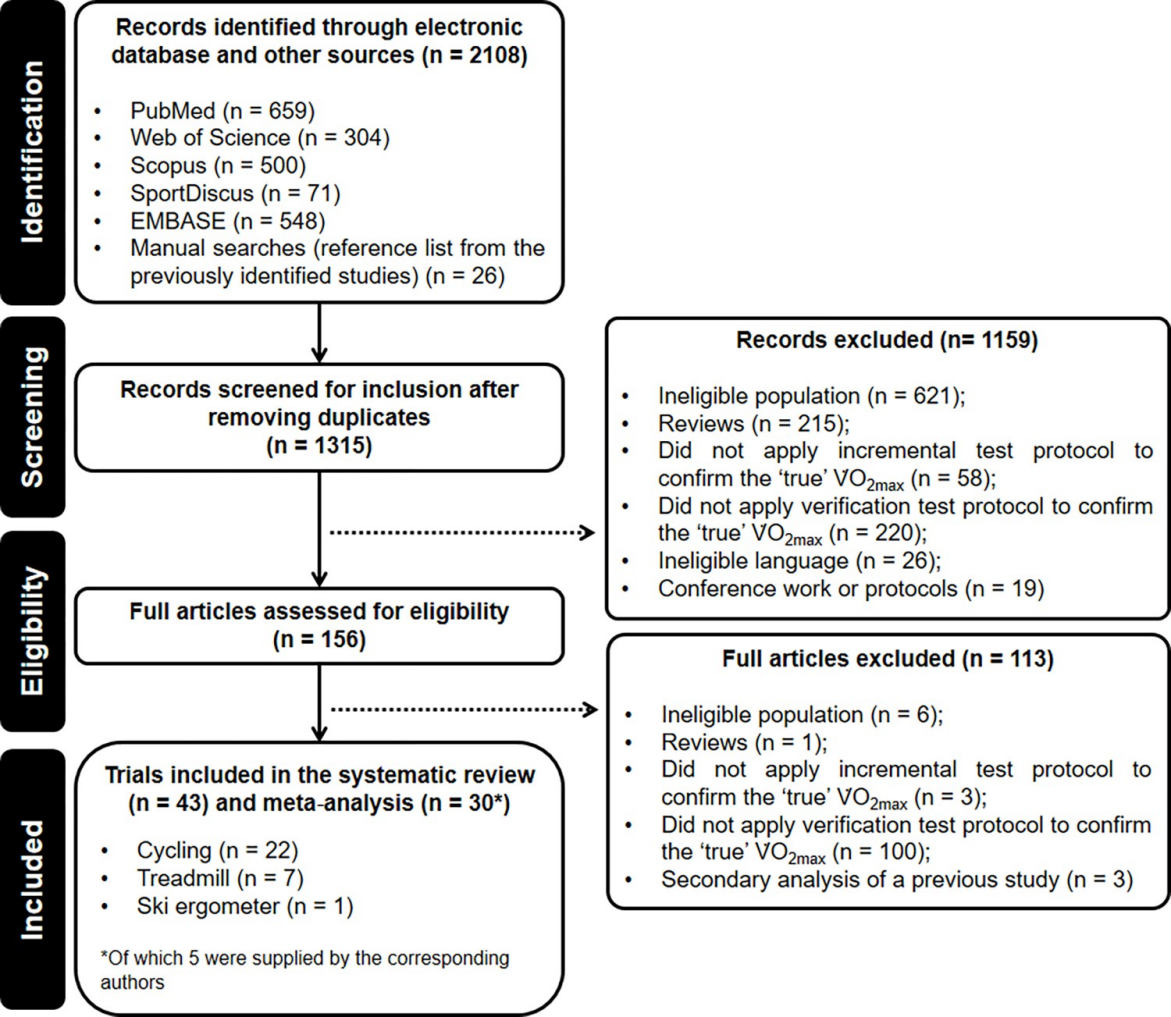
Flowchart for the systematic review and meta-analysis according to PRISMA guidelines. Abbreviations: $\dot{V}O_{2\max}$: maximal oxygen uptake.

Studies were screened for inclusion using a three-step approach: 1) titles and abstracts were initially screened for potentially eligible articles; 2) the full texts of all potentially eligible articles were obtained; and 3) all inclusion and exclusion criteria were applied to full-text articles for the final decision on eligibility. Two of the authors independently determined whether each study met the eligibility criteria. Disagreements were resolved by discussion. Fig 1 shows the screening and selection phases.

### Data extraction and management

The following data were systematically extracted to a Microsoft Excel (2013) spreadsheet: 1) total sample size; 2) characteristics of study participants (special population or clinical condition, sex, age, body mass index, and cardiorespiratory fitness); 3) exercise modality; 4) type of CPET protocol; 5) verification phase protocol and procedures (work rate, type of recovery, timing in relation to whether performed on the same day or different day to the CPET, and whether or not a verification phase threshold criterion was used); and 6) outcome measures (mean ± standard deviation [SD] test duration and absolute $\dot{V}O_{2\max}$ for the CPET and verification phase). Authors of the original articles were contacted to request data when these were not reported. Non-responses from authors were followed up with a second email.

### Quality assessment

The quality of the included studies was independently assessed by two of the authors using a modified version of the Downs and Black checklist [35] (see S2 Fig). Modified versions of this checklist have been employed in reviews in the sport and exercise sciences, which also mainly used cross-sectional studies for their data retrieval [36, 37]. The original checklist comprises 27 items, which are distributed over five sub-scales: reporting (items 1–10), external validity (items 11–13), bias (items 14–20), confounding (items 21–26), and power (item 27) [35]. The Downs and Black checklist was originally designed for intervention studies. Since the present review does not focus on intervention studies, items 8, 9, 12–16, 19, and 22–26 were excluded, and the remaining 14 items included. Furthermore, an additional item was added on whether the included studies provided information on the sampling method. The term “patient” was replaced by “participant”, the term “principal confounders” by “participant characteristics”, and, where applicable, the term “treatment” was interpreted in the context of “testing” [36, 37]. All items, except item numbers 4 and 6, were rated as “Yes” (1 point), “No” (0 points), or “Unknown” (0 points).

For item 4, both the CPET and verification phase needed to be described in sufficient detail, i.e., 1) the duration and magnitude of increments in the CPET; 2) total duration of the CPET; 3) whether the verification phase used a sub- or supra-peak work rate/speed during the CPET; 4) total duration of the verification phase; 5) exercise modality used for the CPET and verification phase; and 6) type and duration of recovery between the CPET and verification phase. Two points were given if all six items were described, and one point was given if four or five of the six items were described. For item 6, both simple outcome data for the major findings of the study, as well as values for $\dot{V}O_{2}$ and its unit of measurement needed to be sufficiently described for this item to be scored a “Yes” with 2 points. Only providing the percentage change or absolute difference in $\dot{V}O_{2}$ between the CPET to exhaustion and verification phase was not sufficient. If only one was sufficiently described, it was rated as a "Yes” with 1 point. A third author helped reach consensus where there were disagreements between the primary reviewers. Quality assessment cut-off points were decided on retrospectively and studies were regarded as low (0–8 points), moderate (9–14 points), or good (15–17 points) methodological quality, based on the total score achieved on the modified Downs and Black checklist. The level of evidence (LoE) for the results of the main and subgroup analyses was categorised from very limited to strong by combining the quality scores of each of the studies included (Table 1). A figure showing the results of the quality assessments was constructed using the R studio programme (R Core Team. R: A language and environment for statistical computing. R foundation for statistical computing Vienna, Austria; 2013).

**Table 1:** Criteria for determining the level of evidence based on the quality of studies included in the main analysis and each of the subgroup analyses (adjusted from the criteria provided by Van Tulder, Furlan [38]).

Level	Criteria
Strong	Data provided in multiple studies of good methodological quality OR in one study of good methodological quality and multiple studies of moderate methodological quality.
Moderate	Data provided in multiple studies of moderate methodological quality OR in one study of good methodological quality.
Limited	Data provided in one study of moderate methodological quality.
Very limited	Data provided in one study of low quality.

### Statistical analysis

All meta-analyses were performed using the Review Manager (RevMan) software version 5.3 (Copenhagen, The Nordic Cochrane Centre, The Cochrane Collaboration, 2014). Data are presented as the mean ± SD, following Cochrane Handbook guidelines [39]. These guidelines state that to perform a meta-analysis of continuous data, authors should utilise the mean value, standard deviation, and number of participants for whom the outcome was measured in each intervention group or test protocol. The outcome was the mean difference (95% confidence interval [CI]) between the CPET and verification phase for the highest absolute $\dot{V}O_{2}$ value in L·min^-1^. Given that absolute $\dot{V}O_{2}$ are continuous data, the weighted mean difference method was used for combining study effect size estimates. With the weighted mean difference method, the pooled effect estimate represents a weighted mean of all included study group comparisons. The weighting assigned to each individual study group (i.e., the comparison of CPET and verification phase results) in the analysis was inversely proportional to the variance of the absolute $\dot{V}O_{2}$. This method typically assigns more weight in the meta-analysis to studies with higher precision (inverse variance) and larger sample sizes. The weighted mean differences were calculated using random-effects models given the study group differences in participants’ characteristics, CPET modalities and protocols, types of recovery, and verification phase protocols. These differences in both participants and protocols characteristics allow that the effect size could vary from study to study. A standardised mean difference method with pooled effect estimate represents a weighted standardised mean of all included study group comparisons and was included as a sensitivity check given the differences in absolute $\dot{V}O_{2}$ observed across different clinical groups.

Heterogeneity of net group changes in absolute $\dot{V}O_{2\max}$ was examined using the Q statistic. Cochran’s Q statistic is computed by summing the squared deviations of each trial’s estimate from the overall meta-analytic estimate and weighting each trial’s contribution in the same manner as in the meta-analysis. The *p*-values were obtained by comparing the statistic with a χ^2^ distribution with *k*-1 degrees of freedom (where *k* is the number of trials). A *p*-value of < 0.10 was adopted since the Q statistic tends to suffer from low differential power [40]. The formal Q statistic was used in conjunction with the methods for assessing heterogeneity. The I^2^ statistic was used to measure the extent of inconsistency among the results of the primary study groups, interpreted approximately as the proportion of total variation in point estimates due to heterogeneity rather than sampling error. Effect sizes with a corresponding I^2^ value of ≤ 50% were considered to have low heterogeneity. Potential publication bias or studies with outlier data were assessed using a funnel plot.

Subgroup analyses were defined a priori to investigate the magnitude of differences between CPET and verification phases due to variations in group characteristics, exercise modality, CPET protocol design, or how the verification phase was performed. The following subgroups based on medical conditions and participant characteristics were considered: paediatric (obese and non-obese under 18 yrs), geriatric (≥ 65 yrs), wheelchair (elite wheelchair athletes and individuals in a wheelchair without spinal cord injury or spina bifida), respiratory (cystic fibrosis, chronic asthma/airway disorders, and bronchiectasis), metabolic (overweight and obese adults with and without metabolic syndrome or hypertension), oncological, and cardiological. Forest plots were constructed to display values at the 95% confidence level. Effect sizes were calculated by subtracting the highest mean values for absolute $\dot{V}O_{2}$ observed in the CPET from the verification phase values, based on grouping studies with selected verification phase characteristics for work rate (i.e., sub vs. supra peak work rate) and type of recovery between the CPET and verification phase (i.e., active vs. passive). The studies were also classified according to whether a criterion threshold for $\dot{V}O_{2\max}$ was used for the verification phase, involving an absolute or relative differences in $\dot{V}O_{2}$ between the CPET and verification phase (i.e., yes vs. no), typically characterised as a percentage difference between tests. In addition to the meta-analytical approach, a participant-level analysis was conducted with data directly reported in the reviewed studies or those supplied by the corresponding authors. The analysis of the differences between the highest $\dot{V}O_{2}$ values elicited in the CPET and verification phase was based on the threshold criteria utilised by the studies or the error of measurement of the metabolic analysers that were used.

## Results

The literature search identified 2108 potential studies, of which 2082 were obtained from electronic databases and 26 from manual searches through a wider inspection of reference lists and citations of these articles. Forty-three articles published between 1993 and 2023 met the eligibility criteria and were included in the systematic review, whilst 30 presented relevant quantitative information to be considered for meta-analysis (see Fig 1).

Tables 2 and 3 show, respectively, the sample characteristics for the reviewed studies and the exercise testing protocols used to measure the $\dot{V}O_{2\max}$ in all participants. Twenty-two studies (51%) used continuous step-incremented protocols, 19 (44%) used ramp-incremented protocols, one used a discontinuous protocol (2%), and one included both continuous and discontinuous step-incremented protocols (2%). Twenty-three studies (53%) used one or more traditional $\dot{V}O_{2\max}$ criteria, of which 17 (39%) used a $\dot{V}O_{2}$ plateau, 16 (37%) used a heart rate plateau or criteria based on the age-predicted maximal heart rate, 17 (39%) used maximal respiratory exchange ratio, 5 (12%) used post-exercise blood lactate concentration, and 6 (14%) used ratings of perceived exertion cut-off values.

**Table 2:** Sample characteristics of the reviewed studies (N = 43).

Study					Mean values		
	Year	Population	Sex	N	Age	BMI	$\dot{V}O_{2\max}$
			M/F		years	kg/m^2^	mL·kg^-1^·min^-1^
Armstrong, Welsman and Winsley [41]	1996	Boys Girls	M F	17 18	9.9	16 20.7	64 52
Astorino, Bediamol [42]	2019	Spinal cord injury	M/F	9/1	33.3	22.6	17.4
Astorino, De La Rosa [43]	2020	Inactive with obesity	F	17	37	39	19.5
Barker, Williams [30]	2011	Children	M/F	8/5	9–10	17.9	50
Baumgart, Moes [44]	2018	Elite para ice hockey players	M/F	14/1	27.1	24.6	36.0
Bhammar, Stickford [45]	2017	Non-obese children Obese children	M/F M/F	5/4 6/3	11 10.9	18.2 29.9	43.4 28.2
Bhammar, Adams-Huet and Babb [46]	2019	Non-obese children Obese children	M/F M/F	20/7 13/13	11.3 11.7	18 28.6	39.8 26.5
Bhammar and Chien [47]	2021	Adults with prehypertension	M F	7 4	21.7 28.3	25.2 23.6	34.6 26.6
Blazquez, Guillamo [48]	2011	Chronic fatigue syndrome	F	32	40.3	24.9	15.6
Bowen, Cannon [49]	2012	Chronic heart failure	M	24	64	29.7	14.6
Causer, Shute [50]	2018	Cystic fibrosis (paediatric) Cystic fibrosis (adults)	M/F M/F	12/5 18/10	12.9 31.3	19.5 22.4	40.1 32.0
Cockcroft, Bond [51]	2019	Adolescents	M	7	14.3	21.5	40.7
de Groot, Takken [52]	2009	Spina bifida (normal ambulation) Spina bifida (community ambulation)	M/F M/F	10 10	9.9 11.1	18.9 21.9	39.4 28.7
Goosey-Tolfrey, Paulson [53]	2014	Elite wheelchair athletes (tetraplegics) Elite wheelchair athletes (paraplegics) Elite wheelchair athletes (without SCI)	M	9 9 8	30 29 27	NR	21.2 39 44.2
Lambrick, Bertelsen [54]	2016	Healthy children	M/F	21/29	9.4	20.6	55.2
Lambrick, Jakeman [55]	2017	Healthy children	M/F	11/10	9.6	20.8	55.3
Leicht, Tolfrey [56]	2013	Elite wheelchair athletes (tetraplegics) Elite wheelchair athletes (paraplegics) Elite wheelchair athletes (without SCI)	M	8 8 8	28.1 31.7 24	NR	23.1 37.1 39.9
Mahoney, Baughman [57]	2019	Obese adults	M	9	24	31.8	35.1
Manresa-Rocamora, Fuertes-Kenneally [58]	2023	Heart failure and reduced ejected fraction	M/F	13/8	64	26	15.0
McCreery, Mackintosh [59]	2021	Bronchiectasis patients	M/F	7/3	64.5	28.3	11.1
Michalski, Ferreira [60]	2022	Stroke patients	M/F	4/3	58	26.5	21.0
Moreno-Cabañas, Ortega [61]	2020	Metabolic syndrome with obesity	M/F	66/34	57.2	32.1	24.6
Moreno-Cabañas, Ortega [62]	2020	Metabolic syndrome with obesity	M/F	28/16	58	31.8	26.2
Murias, Kowalchuk and Paterson [63]	2010	Older adults	F	6	69	27	23.9
Murias, Kowalchuk and Paterson [64]	2010	Older adults	M	8	68	26	28.3
Murias, Pogliaghi and Paterson [65]	2018	Older adults	M	31	68	25.8	33.0
Oliveira, Barker [66]	2019	Adolescents	M	13	14	18.6	50.9
Pal, Schneider [67]	2021	Prostate cancer survivors Breast cancer survivors	M F	10 11	59.9	27.3	23.1
Paulson, Goosey-Tolfrey [68]	2013	Elite wheelchair athletes (tetraplegics) Elite wheelchair athletes (paraplegics) Elite wheelchair athletes (without SCI)	M	8 10 8	31 30 27	NR	21.4 39.4 44.2
Robben, Poole and Harms [69]	2013	Children with expiratory flow limitation	M	12	9.6	18	37.2
Rowland [70]	1993	Healthy children	M/F	6/3	11.4	16.6	55.0
Sansum, Weston [71]	2019	Healthy children	M F	76 52	13.3 13.9	20.4 21.2	46.9 36.2
Sawyer, Tucker [72]	2015	Sedentary with obesity	M F	10 9	33.4 38.4	37.1 34.5	22.0
Saynor, Barker [31]	2013	Cystic fibrosis	M/F	10/4	13.1	24.7	33.0
Saynor, Barker [73]	2013	Cystic fibrosis	M/F	9/4	12.8	21.7	34.8
Schaun, Alberton [32]	2021	Hypertensive older adults	M/F	9/24	67.1	32.2	23.9
Schaun, Alberton [74]	2022	Hypertensive older adults	M/F	4/8	67.3	31.2	25.9
Schneider, Schlüter [75]	2020	Prostate cancer survivors Breast cancer survivors	M F	32 43	66.4 57.5	27.5 25.8	21.7 21.3
Tomlinson, Barker [76]	2018	Cystic fibrosis Healthy children and adolescents	M/F M/F	21/15 21/15	13.4 13.2	20.7 20.2	37.7 39.9
Villanueva, Campbell [77]	2021	Older adults	M F	9 13	69 65	26 26.6	29.8 24.2
Werkman, Hulzebos [78]	2011	Cystic fibrosis	M/F	8/8	14.6	18.1	38.9
Woloschuk, Hodges [79]	2020	Healthy boys	M	12	9.7	16.7	46.9
Wood, Hills [80]	2010	Overweight Obese	M F M F	32 36 35 32	36.7 36.9 37.7 37.1	28.4 28.1 33.6 32.9	42.4 32.2 37.7 27.2

**Abbreviations:** *BMI* = body mass index; *F* = females; *M* = males; *NR* = not reported; *SCI* = spinal cord injury; $\dot{V}O_{2\max}$ = maximal oxygen uptake. *Note*: Authors were contacted to provide unpublished data.

**Table 3:** Characteristics of the cardiopulmonary exercise test (CPET) and verification phase protocols used in the reviewed studies (N = 43).

Study	$\dot{V}O_{2}$ sampling method	Traditional $\dot{V}O_{2\max}$ criteria adopted	Ergometer	CPET Protocol	Recovery Phase	Verification Phase (VP) Protocol	Verification Criteria Threshold
Armstrong, Welsman and Winsley [41]	NS	$\dot{V}O_{2}$ plateau of 150 mL·min^-1^ or 2.1 mL·kg^-1^·min^-1^ during the final minute of the penultimate and final stages; HR ≥ 200 bpm; RER_max_ ≥ 1.00	TR	DiscSI (grade elevation of 2.5% after each 3-min stage, with constant speed)	Different day	↑ slope to 2.5% and 5% in relation to the first test	NS
Astorino, Bediamol [42]	2 x 15-s	$\dot{V}O_{2}$ plateau—individual approach based on the expected O_2_ cost	CYC	Ramp (3 W·min^-1^ for tetraplegics and 13 W·min^-1^ for paraplegics)	10-min active	105% WR_max_	NS
Astorino, De La Rosa [43]	2 x 15-s	HR_max_ ≤ 2 bpm	CYC	Ramp (20 W·min^-1^)	10-min active	105% WR_max_	Typical error score equal to 0.06 L·min^-1^ in $\dot{V}O_{2\max}$ between protocols
Barker, Williams [30]	15-s average	$\dot{V}O_{2}$ plateau (negative linear regression residuals during last 60 s of test); RER_max_ ≥ 1.00; ≥ 85% APMHR and HR_max_ 195 bpm; La_max_ ≥ 6 mmol·L^-1^	CYC	Ramp (10 W·min^-1^)	10-min active and 5-min passive	2-min at 10W, then 105% WR_max_	IP vs. VP: $\dot{V}O_{2\max}$ difference < 5%
Baumgart, Moes [44]	30-s moving average	NS	SKI	CSI (10 W every 30-s)	5-min passive and 3-min active	110% WR_max_	NS
Bhammar, Stickford [45]	20-s	NS	CYC	CSI (10 or 15 W·min^-1^)	15-min passive	2-min at 20W, then 105% WR_max_	Difference between measured $\dot{V}O_{2}$verif and $\dot{V}O_{2\max}$ in relation to the difference in predicted $\dot{V}O_{2}$verif and $\dot{V}O_{2\max}$
Bhammar, Adams-Huet and Babb [46]	20-s	NS	CYC	CSI (10 or 15 W·min-^1^)	15-min passive	105% WR_max_	Difference between measured $\dot{V}O_{2}$verif and $\dot{V}O_{2\max}$ in relation to the difference in predicted $\dot{V}O_{2}$verif and $\dot{V}O_{2\max}$
Bhammar and Chien [47]	20-s	$\dot{V}O_{2}$ plateau (difference between measured $\dot{V}O_{2}$ between the penultimate and final stage was less than 50% of the “expected” increase).	CYC	CSI (20 W·min^-1^ for women and 25 W·min^-1^ for men)	At least 15-min	2-min at 40W (men) or 30W (women), then 105% WR_max_	Difference between measured $\dot{V}O_{2}$verif and expected $\dot{V}O_{2}$verif
Blazquez, Guillamo [48]	NS	NS	CYC	CSI (20 W·min^-1^)	4-min	↑ WR_max_ every 30 sec up to exhaustion	NS
Bowen, Cannon [49]	12-breath rolling average	RER_max_ ≥ 1.10	CYC	Ramp (4–18 W·min^-1^)	5-min	95% WR_max_	$\dot{V}O_{2\max}$ was confirmed when *p* > 0.05 in relation to $\dot{V}O_{2}$verif
Causer, Shute [50]	15-s average	predicted $\dot{V}O_{2peak}$, WR_peak_, HR_max_, 80% VE_max_, RER_max_ 1.03 or 1.05, RPE 9 or 17.	CYC	Ramp (10–25 W·min^-1^)	5-min active and 10-min passive	3-min at 20W, then 110% WR_max_	IP vs. VP: $\dot{V}O_{2\max}$ difference < 9%
Cockcroft, Bond [51]	10-s	NS	CYC	Ramp (details were not reported)	NS	NS	NS
de Groot, Takken [52]	30-s average	$\dot{V}O_{2}$ plateau of 2.1 mL·kg^-1·^min^-1^ (difference between normalised $\dot{V}O_{2peak}$ and $\dot{V}O_{2}$ in the last 30 seconds of the minute before the last minute); RER_max_ ≥ 1.00; > 95% APMHR	TR	CSI (0.25 or 0.5 km·min^-1^ at a constant 2% grade)	4-min	110% WR_max_	NS
Goosey-Tolfrey, Paulson [53]	NS	NS	TR	CSI (grade elevation of 0.1%/40-s and 0.3%·min^-1^ at a constant speed)	5-min active	↑ slope to 0.1 or 0.3% in relation to IP	NS
Lambrick, Bertelsen [54]	10-bins	NS	TR	CSI (0.5 km·h^-1^ at a constant 1% grade)	15-min passive	105% WR_max_	NS
Lambrick, Jakeman [55]	10-s bins	NS	TR	CSI and DiscSI (1 km·h^-1^ each min up to 8 km·h^-1^, then 0.5 km·h^-1^ each min, at a constant 1% grade)	15-min	105% WR_max_	NS
Leicht, Tolfrey [56]	30-s rolling average	$\dot{V}O_{2}$ plateau (negative deviation > 0.1 l/min between observed and predicted peak $\dot{V}O_{2}$ based on linear regression); RER_max_ > 1.05, 1.10, 1.15, and 1.20; > 85%, 90%, 95%, and 100% APMHR and La_max_ > 4, 5, and 6 mmol·L^-1^	TR	CSI (grade elevation of 0.1%/40-s and 0.3%/min at a constant speed)	2-min passive and 5-min active	↑ slope to 0.3 or 0.6% in relation to IP	NS
Mahoney, Baughman [57]	2 x 15-s	NS	CYC	Ramp (W = kg × ($\dot{V}O_{2}$ − 7)/1.8)	Different day	5-min at 50W, then 80, 90, 100 and 105% WR_max_	NS
Manresa-Rocamora, Fuertes-Kenneally [58]	12-breath rolling average	RER_max_ ≥ 1.10	CYC	Ramp (details were not reported)	5-min active	95% WR_max_	IP vs. VP: $\dot{V}O_{2\max}$ difference ≤ 3%
McCreery, Mackintosh [59]	15-s	NS	CYC	Ramp (10 W·min^-1^)	NS	110% WR_max_	IP vs. VP: $\dot{V}O_{2\max}$ difference ≤ 9%
Michalski, Ferreira [60]	30-s	NS	TR	Ramp (predicted initial and final speeds/grades determined during familiarisation)	20-min passive	2-min at 50% WR_max_, 1 min at 70% WR_max_, then 1 stage > IP-WR_max_	NS
Moreno-Cabañas, Ortega [61]	15-s average	$\dot{V}O_{2}$ plateau (difference between measured $\dot{V}O_{2}$ between the penultimate and final stage < 50% of the “expected” increase)	CYC	CSI (15 W·min-^1^ for women and 20 W·min-^1^ for men)	5-min active and 15-min passive	2-min at 30 or 50W, then 110% WR_max_	Difference between $\dot{V}O_{2}$ from CPET vs. $\dot{V}O_{2}$verif ($\dot{V}O_{2}$-work rate relationship) < 50% of the “expected” increase.
Moreno-Cabañas, Ortega [62]	15-s average	$\dot{V}O_{2}$ plateau (difference between measured $\dot{V}O_{2}$ between the penultimate and final stage < 50% of the “expected” increase)	CYC	CSI (15 W·min^-1^ for women and 20 W·min-^1^ for men)	5-min active and 15-min passive	2-min at 30 or 50W, then 110% WR_max_	Difference between $\dot{V}O_{2}$ from CPET vs. $\dot{V}O_{2}$verif ($\dot{V}O_{2}$-work rate relationship) < 50% of the “expected” increase.
Murias, Kowalchuk and Paterson [63]	20-s	NS	CYC	Ramp (12–15 W·min-^1^)	5-min active	85% WR_max_	NS
Murias, Kowalchuk and Paterson [64]	20-s	NS	CYC	Ramp (15–20 W·min-^1^)	5-min active	85% WR_max_	NS
Murias, Pogliaghi and Paterson [65]	20-s average	NS	CYC	Ramp (15–20 W·min-^1^)	5-min active	85% and 105% WR_max_	IP vs. VP: $\dot{V}O_{2\max}$ difference ≤ 2.0 mL·kg^-1^·min^-1^
Oliveira, Barker [66]	NS	NS	TR	CSI (0.5 km/30-s)	10-min	↑ slope to 5%	NS
Pal, Schneider [67]	20-s average	RER_max_ ≥ 1.10; APMHR; La_max_ ≥ 8 mmol·L^-1^; RPE ≥ 18	CYC	CSI (10 W·min-^1^)	10-min passive	110% WR_max_	IP vs. VP: $\dot{V}O_{2\max}$ difference ≤ 3%
Paulson, Goosey-Tolfrey [68]	NS	NS	TR	CSI (grade elevation of 0.1%/40-s and 0.3%/min, at a constant speed)	5-min active	↑ slope to 0.1 or 0.3% in relation to IP	NS
Robben, Poole and Harms [69]	20-s average	$\dot{V}O_{2}$ plateau (difference between modelled and actual > 50% of the regression slope for the linear portion of the $\dot{V}O_{2}$-workrate relationship); RER_max_ ≥ 1.00; ≥ 90% APMHR	CYC	CSI (10 W·min-^1^)	15-min passive	100% WR_max_	Difference between $\dot{V}O_{2}$ from CPET vs. $\dot{V}O_{2}$verif ($\dot{V}O_{2}$-work rate relationship) < 50% of the “expected” increase
Rowland [70]	15-s	$\dot{V}O_{2}$ plateau of < 2 mL·kg-1·min-1	TR	CSI (grade elevation of 2.5% at each 3-min stage, at a constant speed)	Different day	↑ slope to 2.5, 5 and 7% in relation to IP	NS
Sansum, Weston [71]	10 to 15-s average	$\dot{V}O_{2}$ plateau (linear regression over the "linear" portion of the $\dot{V}O_{2}$ response); RER_max_ ≥ 1.00 and 1.1; HR_max_ > 195 bpm, ≥ 85 and 95% APMHR	CYC	Ramp (10–30 W·min-^1^)	25-min	3-min at 20W, then 105 or 110% WR_max_	IP vs. VP: $\dot{V}O_{2\max}$ difference < 5%
Sawyer, Tucker [72]	2 x 15-s average	NS	CYC	Ramp (15 W·min-^1^ for women and 30 W·min-^1^ for men)	5–10 min active	100% WR_max_	≥ 60-s
Saynor, Barker [31]	15-s average	$\dot{V}O_{2}$ plateau (linear regression over the "linear" portion of the $\dot{V}O_{2}$ response); RER_max_ ≥ 1.00 and 1.10; HR_max_ of 180 bpm and ≥ 95% APMHR	CYC	Ramp (10–25 W·min-^1^)	5-min active and 10-min passive	3-min at 20W, then 110% WR_max_	IP vs. VP: $\dot{V}O_{2\max}$ difference < 9%
Saynor, Barker [73]	15-s average	NS	CYC	Ramp (10–25 W·min-^1^)	5-min active and 10-min passive	3-min at 20W, then 110% WR_max_	NS
Schaun, Alberton [32]	2 x 20-s average	$\dot{V}O_{2}$ plateau (difference between measured $\dot{V}O_{2}$ between the penultimate and final stage < 50% of the “expected” increase; 150 mL·min^-1^); HR_max_ within 10 bpm of APMHR; RER_max_ ≥ 1.10; RPE ≥ 18	TR	CSI (0.5 km and 1%/min)	10-min passive	2-min at 50% WR_max_, 1 min at 70% WR_max_, then 1 stage > IP-WR_max_	IP vs. VP: $\dot{V}O_{2\max}$ difference < 3%
Schaun, Alberton [74]	2 x 20-s average	$\dot{V}O_{2}$ plateau of 150 mL·min^-1^; HR_max_ within 10 bpm of APMHR; RER_max_ ≥ 1.10; RPE ≥ 18	TR	CSI (0.5 km and 1%/min)	10-min passive	2-min at 50% WR_max_, 1 min at 70% WR_max_, then 1 stage > IP-WR_max_	IP vs. VP: $\dot{V}O_{2\max}$ difference < 3%
Schneider, Schlüter [75]	20-s average	RER_max_ ≥ 1.1; APMHR; La_max_ ≥ 8 mmol·L^-1^; RPE ≥ 18	CYC	CSI (10 W·min-^1^)	10-min passive	110% WR_max_	IP vs. VP: $\dot{V}O_{2\max}$ difference < 3%
Tomlinson, Barker [76]	10-s	$\dot{V}O_{2}$ plateau (linear regression over the "linear" portion of the $\dot{V}O_{2}$ response); RER_max_ ≥ 1.00 and 1.10; HR_max_ of 180 bpm and ≥ 95% APMHR	CYC	Ramp (no details were reported)	5-min active and 10-min passive	3-min at 20W, then 110% WR_max_	NS
Villanueva, Campbell [77]	3 x 10-s	NS	CYC	Ramp (15 W·min-^1^ for women and 20 W·min-^1^ for men)	10-min active	85 and 110% WR_max_	NS
Werkman, Hulzebos [78]	30-s	$\dot{V}O_{2}$ plateau of ≤ 2.1 mL·kg-^1^·min^-1^; RER_max_ ≥ 1.00; HR_max_ ≥ 95% APMHR	CYC	CSI (10, 15 or 20 W·min-^1^ based on each participant’s height)	10-min passive	1-min unloaded cycling, then an increase in workload every 10-s	NS
Woloschuk, Hodges [79]	2 x 15-s	NS	CYC	CSI (10–15 W·min-^1^)	10-min	105% WR_max_	NS
Wood, Hills [80]	30-s average	$\dot{V}O_{2}$ plateau < 50% of expected for the change in WR; HR within ± 11 bpm of APMHR; RER_max_ ≥ 1.15; La_max_ ≥ 8 mmol·L^-1^; RPE ≥ 18	TR	CSI (grade elevation of 2.5%/min at a constant speed)	5–10 min passive	↑ WR_max_ every minute up to exhaustion	NS

**Abbreviations:** *APMHR* = age-predicted maximal heart rate; *bpm* = beats per minute; *CPET* = cardiopulmonary exercise test; *CSI* = continuous step-incremented; *CYC* = cycling; *DiscSI* = discontinuous step-incremented; *HR* = heart rate; *HR*_*max*_ = maximal heart rate; *IP* = incremental phase; *kg* = kilogram; *La*_*max*_ = maximal blood lactate concentration; *NS* = not stated; *O*_*2* =_ *oxygen; RER*_*max*_ = maximal respiratory exchange ratio; *RPE* = rating of perceived exertion; *SD* = standard deviation; *SKI* = ski ergometer; *TR* = treadmill; $\dot{V}O_{2}$ = oxygen uptake; $\dot{V}O_{2\max}$ = maximal oxygen uptake; $\dot{V}O_{2}$*verif =* maximal oxygen uptake obtained in the verification phase*; VP* = verification phase; *W* = watts; *WR* = work rate; *WR*_*max*_ = maximal work rate. *Note*: Authors were contacted to provide unavailable data.

Regarding respiratory expired gas analysis procedures, smoothing of pulmonary gas exchange data is required during exercise testing for the determination of $\dot{V}O_{2\max}$, especially for data collected from participants on a breath-by-breath basis. The most common approach was based on time averages. Thirty-two studies (74%) reported using time averages of between 10 and 30 s, two (5%) used moving time averages, two (5%) applied 12-breath rolling averages, and three (7%) did not describe which $\dot{V}O_{2}$ data processing method was applied. Amongst more traditional expired gas collection techniques, four studies (9%) used Douglas bag collections of between 30 and 60 s. No study addressed the effect of different $\dot{V}O_{2}$ sampling intervals on the difference between the peak $\dot{V}O_{2}$ values attained in the CPET and verification phase.

Regarding the type of recovery between CPET termination and the start of the verification phase, 10 studies (23%) used active recovery, 11 (26%) used passive recovery, 8 (19%) adopted a combination of passive and active recovery, and 11 (26%) did not report the type of recovery. The verification phase was carried out on a different day as the CPET in three studies (7%). When the verification phase was performed on the same day as the CPET, the recovery period varied from 4 to 25 min. Ten studies (23%) used a 10-min recovery, which was the most common. Two articles (5%) did not state the duration of the recovery period used in the studies.

Twenty-five studies (58%) used square-wave verification phase protocols (i.e., the work rate was immediately increased to the target sub or supra peak work rate), whereas 17 (40%) used multistage verification phase protocols characterised by an initial warm-up stage. Only one study (2%) did not describe the verification phase protocol. The peak work rate used in the verification phase protocols ranged from 80% to 110% of the peak work rate attained in the CPET across studies. Most studies applied a supra peak work rate based on the peak work rate achieved in the CPET (n = 33; 77%). Three studies (7%) applied both sub and supra peak work rates within the same study, two (5%) used peak work rate, four (9%) applied only a sub peak work rate, and one (2%) did not describe the verification phase work rate. The mean times to exhaustion for the CPET and verification phase were 568 s (SD, 143 s) and 127 s (SD, 57 s), respectively.

Twenty-two studies (51%) employed threshold criteria to analyse differences between the highest $\dot{V}O_{2}$ attained in the CPET and verification phase and were frequently based on the intra-subject coefficient of variation acquired from the researchers’ laboratories or from published literature. Threshold criteria included a difference in $\dot{V}O_{2}$ (L·min^-1^) of < 2%, < 3%, < 5% and < 9%, and an absolute difference between measured and predicted $\dot{V}O_{2}$ from linear extrapolations of $\dot{V}O_{2}$ / work rate responses during the CPET (such as < 50% of the “expected” increase). Other cut-off points included a typical error of 0.06 L·min^-1^, 2.1 mL·kg^-1^·min^-1^, or when the comparison of the highest group mean $\dot{V}O_{2}$ value obtained in the CPET versus the verification phase resulted in *p* > 0.05.

### Methodological quality of the included studies

**Fig 2:**
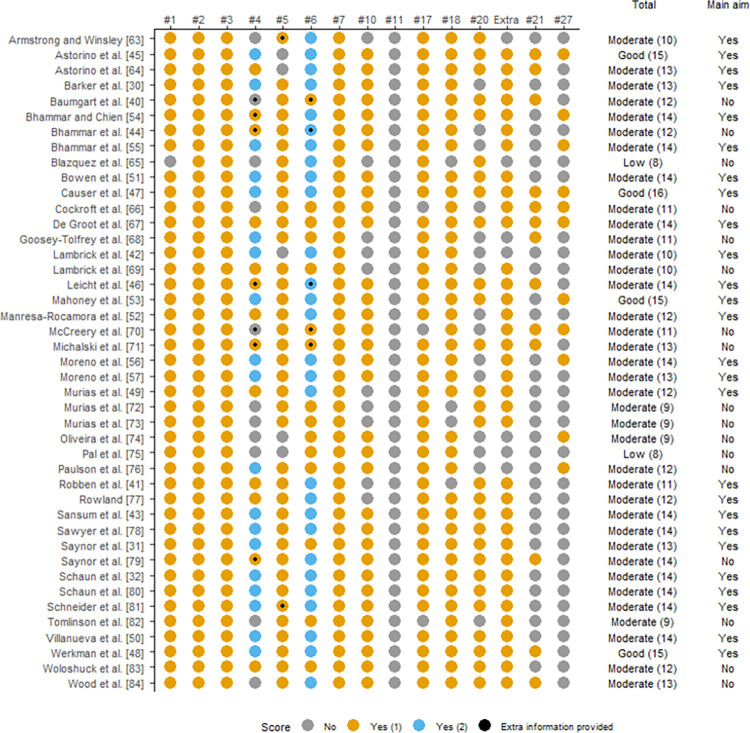
Quality scores for the 43 included studies. Grey dots for items scored as ‘no’, yellow dots are for items scored ‘yes’ (1), and blue dots for ‘yes’ (2). Black dots are added where authors retrospectively provided extra information that would have led to a higher quality appraisal score if included in the original publication.

There was 86% agreement between the two authors in ranking the items initially, and full agreement was reached upon discussion with a third author. Two studies (5%) were rated as having low methodological quality, 37 (86%) as moderate, and four (9%) as good (Fig 2). The quality of the studies included in the main and subgroup comparisons were used to underpin the respective LoE.

### Group-level quantitative data synthesis: Differences between the highest $\dot{V}O_{2}$ attained in the CPET and verification phase

**Fig 3:**
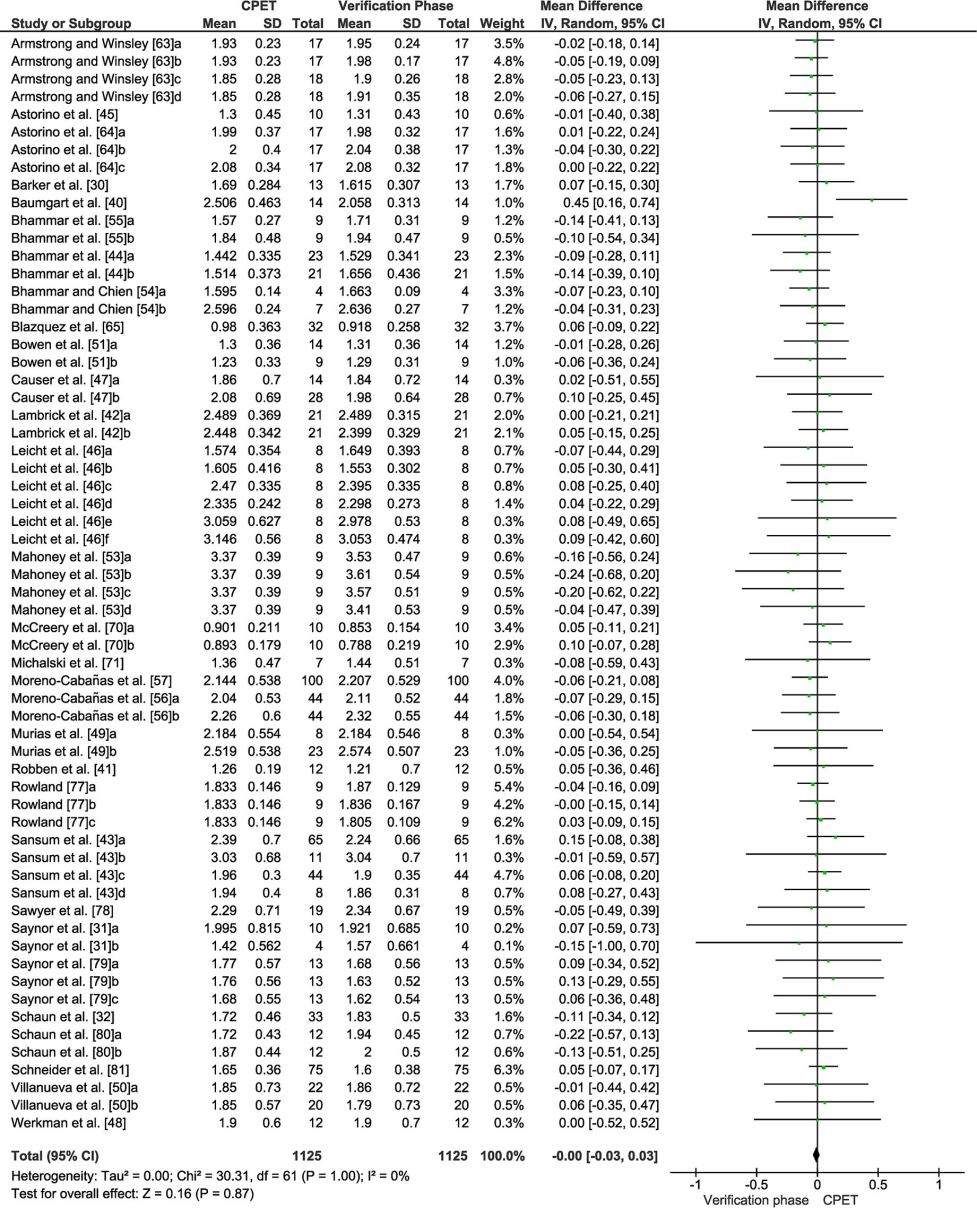
Forest plot for all the studies included in the meta-analysis (n = 30) for the highest $\dot{V}O_{2}$ (L·min^-1^) attained in the cardiopulmonary exercise test (CPET) and verification phase using a random-effects model. Data are reported as mean differences adjusted for control data (95% CI).

**Fig 4:**
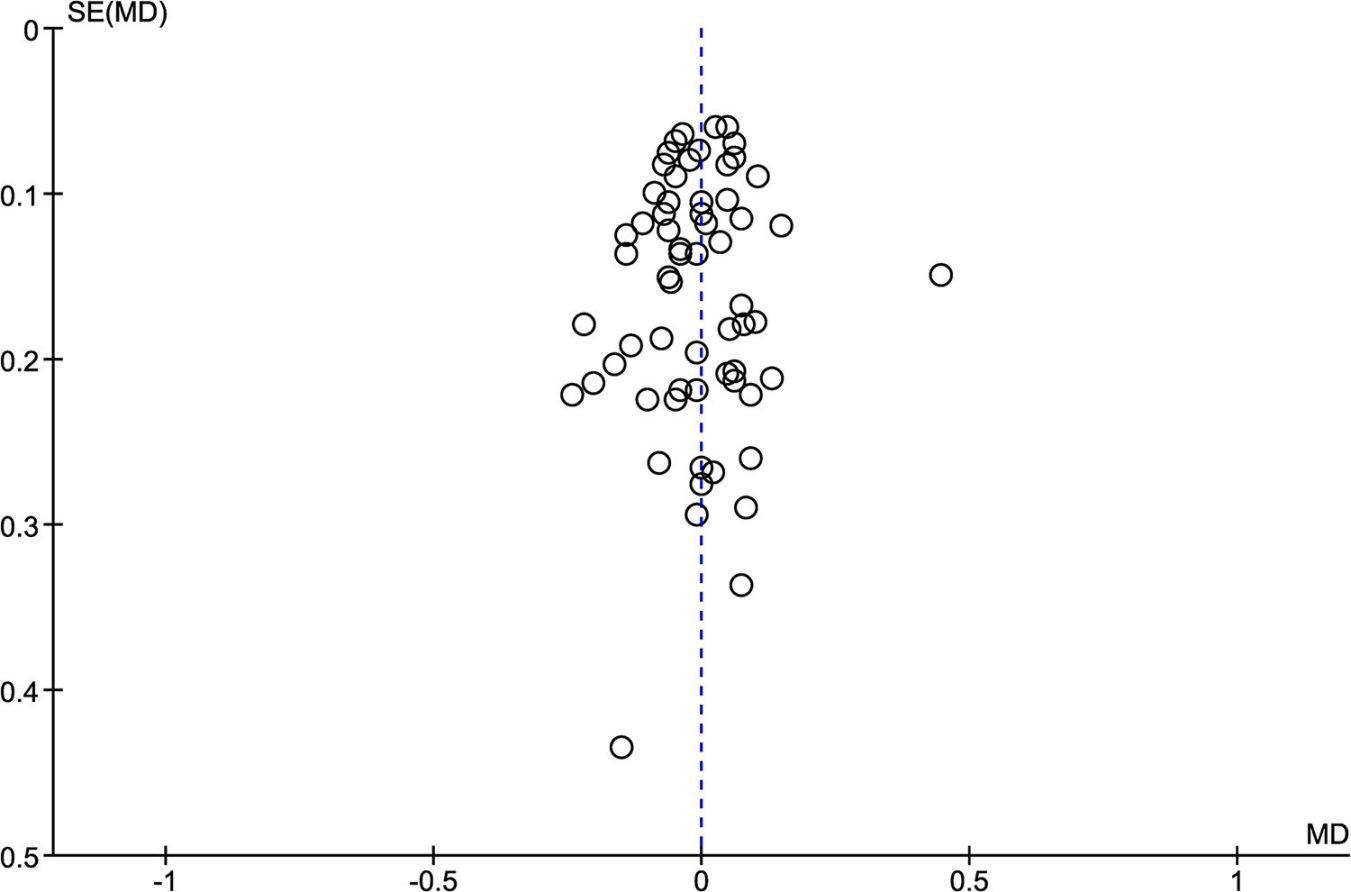
Funnel plot assessment of publication bias for the highest $\dot{V}O_{2}$ (L·min^-1^) attained in the cardiopulmonary exercise test (CPET) and verification phase. One outlier identified, which may relate to methodological error within the verification phase protocol (see discussion section).

Table 4 shows comparisons between the highest $\dot{V}O_{2}$ values elicited in the CPET and verification phase for each study. Fig 3 displays the forest plot of effect sizes and 95% CIs for the highest $\dot{V}O_{2}$ values (30 studies) based on the random effects meta-analysis results. The highest $\dot{V}O_{2}$ was not different between CPET and verification phase (mean difference = -0.00 [95% CI = -0.03 to 0.03] L·min^-1^, *p* = 0.87; LoE: strong). Given the potential for large heterogeneity in mean $\dot{V}O_{2\max}$ values across the different populations included in the review, the overall and subgroup meta-analysis findings were robust to a sensitivity check using the alternative statistical approach of using standardised mean differences. This method resulted in a different weighting pattern to the individual study group differences between the CPET and verification phase $\dot{V}O_{2\max}$ values (data not presented). However, the overall effect was unchanged (standardised mean difference = -0.01 [95% CI = -0.09 to 0.08] L·min^-1^, *p* = 0.87, LoE: strong). Pooled data for $\dot{V}O_{2\max}$ following the CPET and verification phase showed no significant heterogeneity among all the studies (see Fig 3). Except for one of the included studies judged to be an outlier [44], the meta-analysed studies were judged to have a low-risk of bias as shown by the funnel plot (Fig 4).

**Table 4:** Overall group-level comparisons for the reviewed studies for the highest $\dot{V}O_{2}$ values attained in the cardiopulmonary exercise test (CPET) and verification phase (VP) (N = 30).

Study	Specific Experimental Condition	CPET			VP			% Weight	Mean Difference
		Mean L·min^-1^	SD L·min^-1^	n	Mean L·min^-1^	SD L·min^-1^	n		IV, Random, 95% CI [L·min^-1^]
Armstrong, Welsman and Winsley [41]	↑ slope to 2.5% in relation to the first test (boys) ↑ slope to 5% in relation to the first test (boys)	1.93 1.93	0.23 0.23	17 17	1.95 1.98	0.24 0.17	17 17	3.5% 4.8%	-0.02 [-0.18, 0.14] -0.05 [-0.19, 0.09]
	↑ slope to 2.5% in relation to the first test (girls) ↑ slope to 5% in relation to the first test (girls)	1.85 1.85	0.28 0.28	18 18	1.90 1.91	0.26 0.35	18 18	2.8% 2.0%	-0.05 [-0.23, 0.13] -0.05 [-0.27, 0.15]
Astorino, Bediamol [42]	N/A	1.30	0.45	10	1.31	0.43	10	0.6%	-0.01 [-0.40, 0.38]
Astorino, De La Rosa [43]	Baseline Post 3-week Post 6-week Post 6-week	1.99 2.00 2.08	0.37 0.40 0.34	17 17 17	1.98 2.04 2.08	0.32 0.38 0.32	17 17 17	1.6% 1.3% 1.8%	0.01 [-0.22, 0.24] -0.04 [-0.30, 0.22] 0.00 [-0.22, 0.22]
Barker, Williams [30]	N/A	1.69	0.284	13	1.615	0.307	13	1.7%	0.07 [-0.15, 0.30]
Baumgart, Moes [44]	N/A	2.506	0.463	14	2.058	0.313	14	1.0%	0.45 [0.16, 0.74]
Bhammar, Stickford [45]	Obese children Non-obese children	1.57 1.84	0.27 0.48	9 9	1.71 1.94	0.31 0.47	9 9	1.2% 0.5%	-0.14 [-0.41, 0.13] -0.10 [-0.54, 0.34]
Bhammar, Adams-Huet and Babb [46]	Obese children Non-obese children	1.514 1.442	0.373 0.335	21 23	1.656 1.529	0.436 0.341	21 23	1.5% 2.3%	-0.14 [-0.39, 0.10] -0.09 [-0.28, 0.11]
Bhammar and Chien [47]	Women Men	1.595 2.596	0.14 0.24	4 7	1.663 2.636	0.09 0.27	4 7	3.3% 1.2%	-0.07 [-0.23, 0.10] -0.04 [-0.31, 0.23]
Blazquez, Guillamo [48]	N/A	0.98	0.363	32	0.918	0.258	32	3.7%	0.06 [-0.09, 0.22]
Bowen, Cannon [49]	$\dot{V}O_{2\max}$ $\dot{V}O_{2peak}$	1.30 1.23	0.36 0.33	14 9	1.31 1.29	0.36 0.31	14 9	1.2% 1.0%	-0.01 [-0.28, 0.26] -0.06 [-0.36, 0.24]
Causer, Shute [50]	Paediatric Adults	1.86 2.08	0.70 0.69	14 28	1.84 1.98	0.72 0.64	14 28	0.3% 0.7%	0.02 [-0.51, 0.55] 0.10 [-0.25, 0.45]
Lambrick, Jakeman [55]	CSI DisCSI	2.489 2.448	0.369 0.342	21 21	2.489 2.399	0.315 0.329	21 21	2.0% 2.1%	0.00 [-0.21, 0.21] 0.05 [-0.15, 0.25]
Leicht, Tolfrey [56]	Tetraplegics (Day 1) Tetraplegics (Day 2) Paraplegics (Day 1) Paraplegics (Day 2) Non-SCI (Day 1) Non-SCI (Day 2)	1.574 1.605 2.47 2.335 3.059 3.146	0.354 0.416 0.335 0.242 0.627 0.56	8 8 8 8 8 8	1.649 1.553 2.395 2.298 2.978 3.053	0.393 0.302 0.335 0.273 0.53 0.474	8 8 8 8 8 8	0.7% 0.7% 0.8% 1.4% 0.3% 0.3%	-0.07 [-0.44, 0.29] 0.05 [-0.30, 0,41] 0.08 [-0.25, 0,40] 0.04 [-0.22, 0.29] 0.08 [-0.49, 0.65] 0.09 [-0.42, 0.60]
Mahoney, Baughman [57]	80% WR_max_ 90% WR_max_	3.37 3.37	0.39 0.39	9 9	3.53 3.61	0.47 0.54	9 9	0.6% 0.5%	-0.16 [-0.56, 0.24] -0.24 [-0.68, 0.20]
	100% WR_max_ 105% WR_max_	3.37 3.37	0.39 0.39	9 9	3.57 3.41	0.51 0.53	9 9	0.5% 0.5%	-0.20 [-0.62, 0.22] -0.04 [-0.47, 0.39]
McCreery, Mackintosh [59]	Baseline Post 8-week	0.901 0.893	0.211 0.179	10 10	0.853 0.788	0.31 0.47	10 10	3.4% 2.9%	0.05 [-0.11, 0.21] 0.10 [-0.07, 0.28]
Michalski, Ferreira [60]	N/A	1.36	0.47	7	1.44	0.51	7	0.3%	-0.08 [0.59, 0.43]
Moreno-Cabañas, Ortega [61]	N/A	2.144	0.538	100	2.207	0.529	100	4.0%	-0.06 [-0.21, 0.08]
Moreno-Cabañas, Ortega [62]	Pre-training Post-training	2.04 2.26	0.53 0.6	44 44	2.11 2.32	0.52 0.55	44 44	1.8% 1.5%	-0.07 [-0.29, 0.15] -0.06 [-0.30, 0.18]
Murias, Pogliaghi and Paterson [65]	85% WR_max_ 105% WR_max_	2.184 2.519	0.554 0.538	8 23	2.184 2.574	0.546 0.507	8 23	0.3% 1.0%	0.00 [-0.54, 0.54] -0.05 [-0.36, 0.25]
Robben, Poole and Harms [69]	Boys	1.26	0.19	12	1.12	0.7	12	0.5%	0.05 [-0.36, 0.46]
Rowland [70]	↑ slope to 2.5% in relation to the first test ↑ slope to 5% in relation to the first test ↑ slope to 7.5% in relation to the first test	1.833 1.833 1.833	0.146 0.146 0.146	9 9 9	1.87 1.836 1.805	0.129 0.167 0.109	9 9 9	5.4% 4.2% 6.2%	-0.04 [-0.16, 0.09] -0.00 [-0.15, 0.14] 0.03 [-0.09, 0.15]
Sansum, Weston [71]	Non-overweight boys Overweight boys Non-overweight girls Overweight girls	2.39 3.03 1.96 1.94	0.70 0.68 0.30 0.40	65 11 44 8	2.24 3.04 1.90 1.86	0.66 0.70 0.35 0.31	65 11 44 8	1.6% 0.3% 4.7% 0.7%	0.15 [-0.08, 0.38] -0.01 [-0.59, 0.57] 0.06 [-0.08, 0.20] 0.08 [-0.27, 0.43]
Sawyer, Tucker [72]	N/A	2.29	0.71	19	2.34	0.67	19	0.5%	-0.05 [-0.49, 0.39]
Saynor, Barker [31]	Boys Girls	1.995 1.42	0.815 0.562	10 4	1.921 1.57	0.685 0.661	10 4	0.2% 0.1%	0.07 [-0.59, 0.73] -0.15 [-1.00, 0.70]
Saynor, Barker [73]	Test 1 Test 2 Test 3	1.77 1.76 1.68	0.57 0.56 0.55	13 13 13	1.68 1.63 1.62	0.56 0.52 0.64	13 13 13	0.5% 0.5% 0.5%	0.09 [-0.34, 0.52] 0.13 [-0.29, 0.55] 0.06 [-0.36, 0.48]
Schaun, Alberton [32]	N/A	1.72	0.46	33	1.83	0.50	33	1.6%	-0.11 [-0.34, 0.12]
Schaun, Alberton [74]	Baseline Post 12-week	1.72 1.87	0.43 0.44	12 12	1.94 2.00	0.45 0.50	12 12	0.7% 0.6%	-0.22 [-0.57, 0.13] -0.13 [-0.51, 0.25]
Schneider, Schlüter [75]	N/A	1.65	0.36	75	1.60	0.38	75	6.3%	0.05 [-0.07, 0.17]
Villanueva, Campbell [77]	85% WR_max_ 110% WR_max_	1.85 1.85	0.73 0.57	22 20	1.86 1.79	0.72 0.73	22 20	0.5% 0.5%	-0.01 [-0.44, 0.42] 0.06 [-0.35, 0.47]
Werkman, Hulzebos [78]	N/A	1.90	0.60	12	1.90	0.70	12	0.3%	0.00 [-0.52, 0.52]

**Abbreviations:** *CI* = confidence interval; *CPET* = cardiopulmonary exercise test; *CSI* = continuous step-incremented; *DisCSI* = discontinuous step-incremented; *IV* = inverse variance; *n* = total of participants included; *Non-SCI* = non-spinal cord-injured; *N/A* = not applicable; *SD* = standard deviation; $\dot{V}O_{2peak}$ = peak oxygen uptake; $\dot{V}O_{2\max}$ = maximal oxygen uptake; $\dot{V}O_{2peak}$ = peak oxygen uptake; *VP* = verification phase; *WR*_*max*_ = maximal work rate. Note: whenever possible, authors were contacted to provide unavailable data. %Weight = weight attributed to each study due to its statistical power.

There was no statistically significant difference between CPET and verification-derived $\dot{V}O_{2\max}$ for the paediatric group that included seven studies with 18 experimental conditions (mean difference = -0.01 [95% CI = -0.05 to 0.03] L·min^-1^, *p* = 0.68, LoE: moderate). Additionally, the subgroup analysis of obese and non-obese paediatric participants, composed of two studies with four experimental conditions, revealed no statistically significant difference (mean difference = -0.11 [95% CI = -0.24 to 0.01] L·min^-1^, *p* = 0.08, LoE: moderate). The wheelchair group consisted of three studies with eight experimental conditions, and no statistically significant difference was observed between the CPET and verification phase (mean difference = 0.11 [95% CI = -0.02 to 0.23] L·min^-1^, *p* = 0.10, LoE: strong). The chronic respiratory group consisted of 6 studies and 11 experimental conditions that demonstrated no statistically significant significance between the CPET and verification phase (mean difference = 0.07 [95% CI = -0.02 to 0.17] L·min^-1^, *p* = 0.14, LoE: strong). The 15 girls from the study by Robben et al. [69] were removed from the meta-analysis, since the reported SD was an extreme outlier (e.g., the study would have been weighted at 40.7% in the final meta-analysis). The subgroup of paediatric patients with cystic fibrosis included four studies and seven experimental conditions (mean difference = 0.06 [95% CI = -0.13 to 0.25] L·min^-1^, *p* = 0.55 LoE: strong). The geriatric group incorporated four studies with seven experimental conditions, and results showed no statistically significant difference between the CPET and verification phase (mean difference of -0.08 [95% CI = -0.21 to 0.05] L·min^-1^, *p* = 0.20 LoE: moderate). Finally, the metabolic group including individuals with overweight or obesity (with and without metabolic disease), comprised six studies and 13 experimental conditions. These studies used ramp-based cycle ergometry and demonstrated no statistically significant difference between CPET and the verification phase (mean difference = -0.06 [95% CI = -0.13 to -0.01] L·min^-1^, *p* = 0.09).

**Fig 5:**
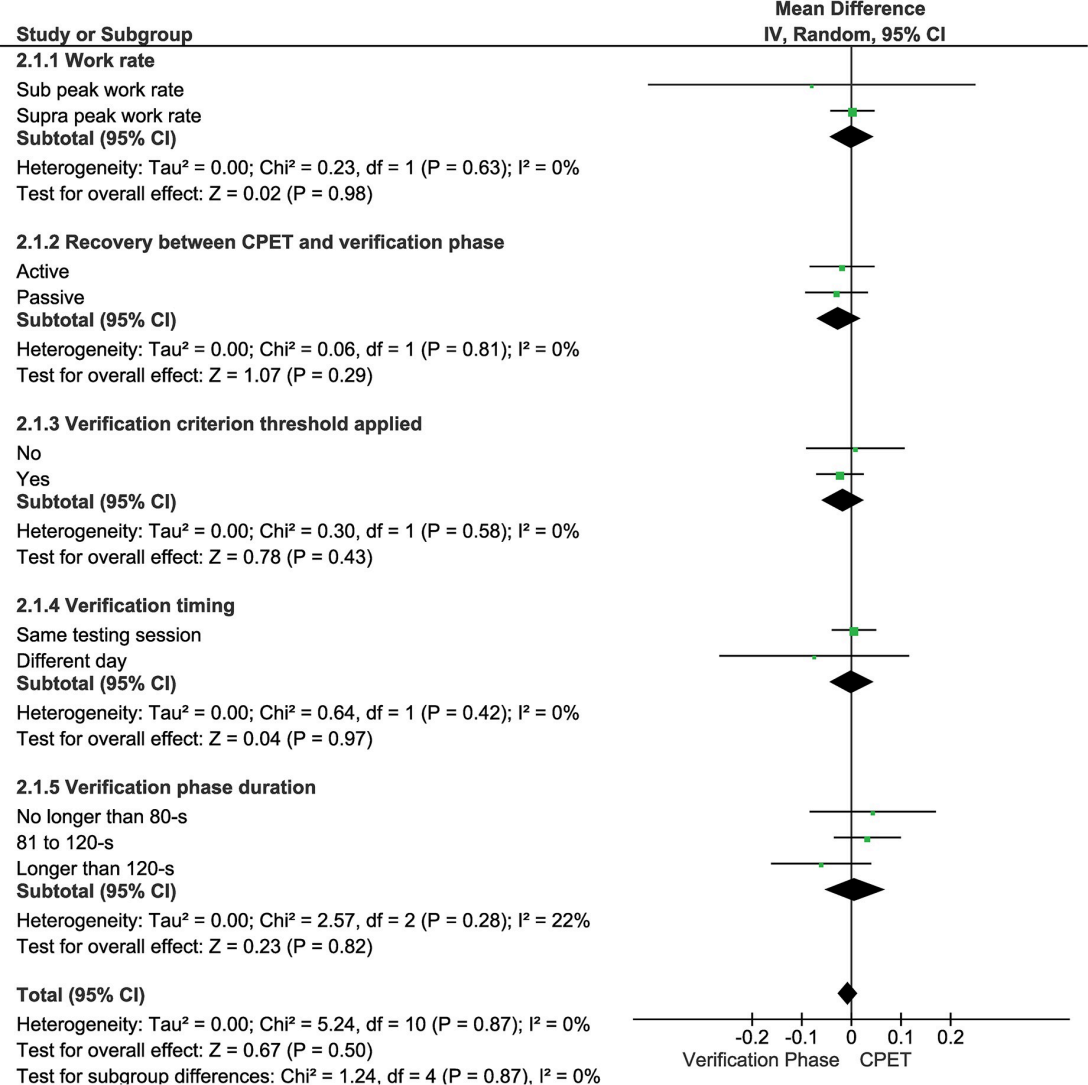
Mean differences (95% confidence intervals [CIs]) between the highest $\dot{V}O_{2}$ values (L·min^-1^) in the cardiopulmonary exercise test (CPET) and verification phase according to the verification-phase characteristics for work rate (i.e., sub WR_peak_ vs. supra WR_peak_), recovery (i.e., active vs. passive), adoption of a criterion threshold (i.e., yes vs. no), timing (performed on the same day vs. a different day to the CPET), and duration (i.e., no longer than 80 s, from 81 to 120-s and longer than 120-s).

Regarding the CPET and verification phase protocols (Fig 5 combines all subgroup analyses into the same category), there were no statistically significant differences for verification phase work rate (mean difference = 0.00 [95% CI = -0.04 to 0.04] L·min^-1^, *p* = 0.98, LoE: strong), recovery mode (mean difference = -0.02 [95% CI = -0.07 to 0.02] L·min^-1^, *p* = 0.29, LoE: strong), adoption of verification phase criteria (mean difference = -0.02 [95% CI = -0.06 to 0.03] L·min^-1^, *p* = 0.43, LoE: strong), verification phase performed on the same or on a separate day as the CPET (mean difference = 0.00 [95% CI = -0.04 to 0.04] L·min^-1^, *p* = 0.97, LoE: strong), or verification phase duration (mean difference = 0.01 [95% CI -0.05 to 0.07] L·min^-1^, *p* = 0.82, LoE: strong). All combined categories were not statistically significantly different when analysed separately (*p* = 0.28 to *p* = 0.93, LoE: strong). The exercise mode used did not influence the results for treadmill (mean difference = -0.02 [95% CI -0.07 to 0.03] L·min^-1^, *p* = 0.41, LoE: moderate) or cycling (mean difference = 0.00 [95% CI -0.04 to 0.04 L·min^-1^, *p* = 0.93, LoE: strong) across all included studies. The highest $\dot{V}O_{2}$ attained in the CPET was similar among protocols and their subsequent verification phase (*p* = 0.30; *p* = 0.43 and *p* = 0.56) for ramp, discontinuous, and continuous step-incremented protocols, respectively; LoE: moderate to strong.

### Participant-level analysis of the highest $\dot{V}O_{2}$ values attained in the CPET and verification phase

**Fig 6:**
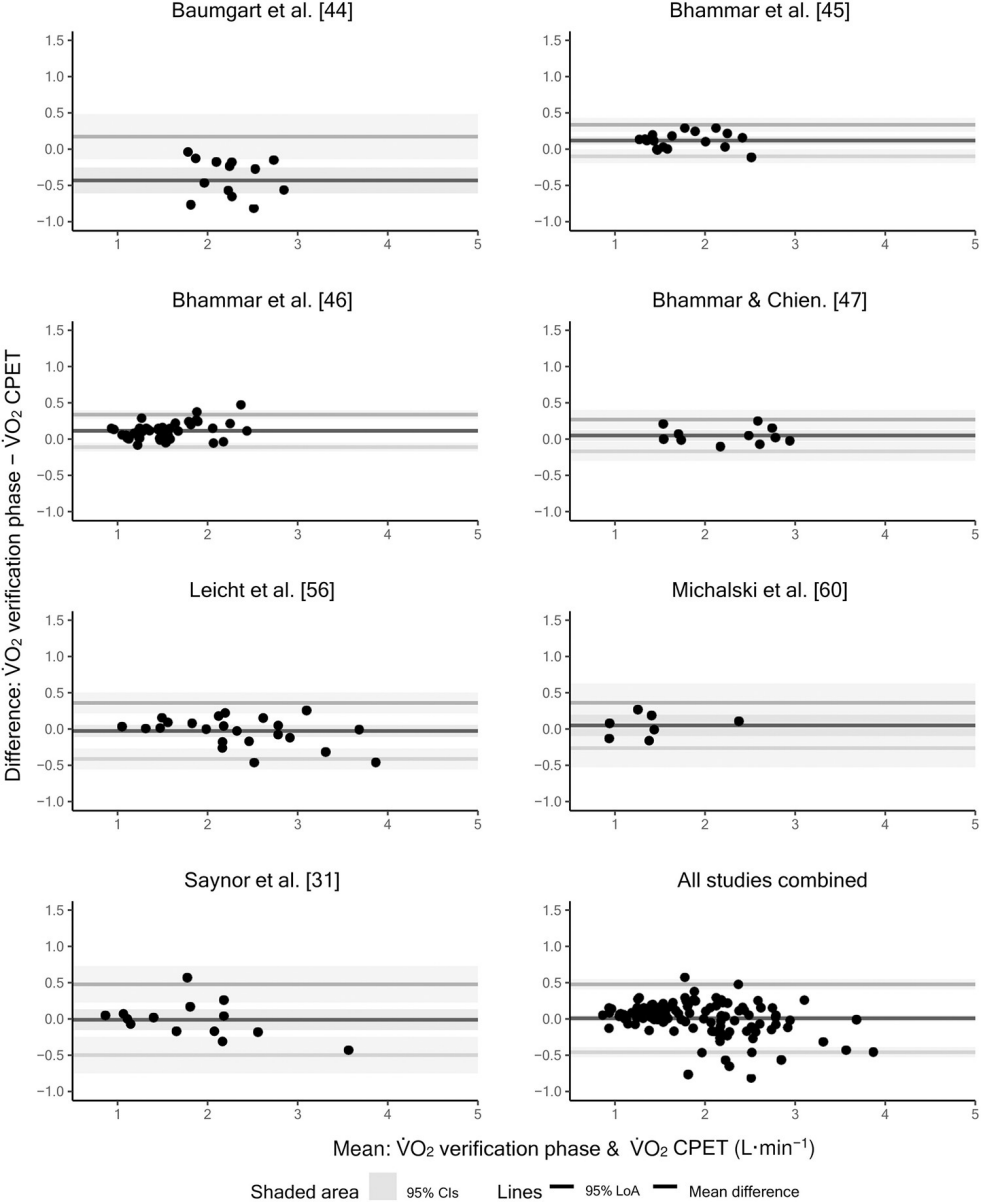
Participant-level differences between the highest $\dot{V}O_{2}$ values obtained in the cardiopulmonary exercise test (CPET) and verification phase for the seven studies where these data were available.

Only 23 (53.5%) of the 43 reviewed studies reported how many participants achieved a lower, equal, or higher $\dot{V}O_{2}$ value in the verification phase versus the CPET or supplied participant-level $\dot{V}O_{2}$ data from which this information could be obtained. Table 5 shows the percentages of participants that achieved a lower, equal, or higher $\dot{V}O_{2}$ value in the verification phase versus the CPET for each study where this information was available. Fig 6 shows participant-level differences between the highest $\dot{V}O_{2}$ values obtained in the CPET and verification phase for the seven studies where these data were available [31, 44–47, 56, 60].

**Table 5:** Frequencies and percentages for whether the verification phase (VP) elicited a lower, similar, or higher $\dot{V}O_{2}$ than the cardiopulmonary exercise test (CPET) for the 23 studies that mentioned or reported this information. Participant-level data were supplied by the authors for the first seven studies in the table.

Study	Population characteristics and sample size	Highest $\dot{V}O_{2}$ lower, similar, or higher in VP versus CPET?		
		Lower	Similar	Higher
Baumgart, Moes [44]^a^	Para ice hockey athletes (n = 14)	100%	0%	0%
Bhammar, Stickford [45]^b^	Obese children (n = 9) and non-obese children (n = 9)	NR	NR	66.7%
Bhammar, Adams-Huet and Babb [46]^b^	Obese children (n = 21) and non-obese children (n = 23)	NR	NR	NR
Bhammar and Chien [47]^c^	Adults with prehypertension (n = 11)	NR	NR	27.3%
Leicht, Tolfrey [56]	Wheelchair athletes (n = 24)	33.3%	29.2%	37.5%
Michalski, Ferreira [60]	Stroke patients (n = 7)	28.5%	14.4%	57.1%
Saynor, Barker [31]	Cystic fibrosis (n = 14)	21.4%	57.1%	21.4%
Astorino, De La Rosa [43]	Inactive with obesity (n = 17)	NR	NR	30%, 53% and 41% (baseline, post 3 and 6 week)
Bowen, Cannon [49]	Heart failure patients (n = 24)	20.8%	58.4%	20.8%
Causer, Shute [50]	Cystic fibrosis (paediatric n = 14 and adults n = 28)	14.3% 3.6%	64.3% 75%	21.4% 21.4%
de Groot, Takken [52]	Spina bifida (n = 20)	10%	65%	25%
Lambrick, Jakeman [55]	Healthy children (n = 21)	NR	NR	38% (CSI) and 19% (DSI)
Mahoney, Baughman [57]	Adults with obesity (n = 9)	NR	NR	88.9%
Manresa-Rocamora, Fuertes-Kenneally [58]	Heart failure patients (n = 21)	23.8%	52.4%	23.8%
Moreno-Cabañas, Ortega [61]	Metabolic syndrome adults with obesity (n = 100)	0%	60%	40%
Moreno-Cabañas, Ortega [62]	Metabolic syndrome adults with obesity (n = 44)	0%	59%	41%
Sansum, Weston [71]	Healthy children (n = 128)	0%	88%	12%
Sawyer, Tucker [72]	Adults with obesity (n = 19)	NR	NR	68.4%
Schaun, Alberton [32]	Older adults with hypertension (n = 33)	21.2%	24.2%	54.6%
Schaun, Alberton [74]	Older adults with hypertension (n = 12)	8.3% baseline 16.7% post 12-week	16.7% baseline and 8.3% post 12-week	75% baseline and post 12-week
Schneider, Schlüter [75]	Cancer survivors (n = 75)	0%	68%	32%
Villanueva, Campbell [77]	Older adults (n = 22 sub peak and n = 20 supra peak verification phase)	14% sub peak 35% supra peak	68% sub peak 40% supra peak	18% sub peak 25% supra peak
Wood, Hills [80]	Overweight and obese adults (n = 114^d^)	NR	45.6%	NR

^a^ 2% threshold criterion used to decide whether the highest $\dot{V}O_{2}$ was similar in the CPET and verification phase, which was based on the inherent variability of the metabolic cart

^b^ Participant-level data were provided but the missing frequencies in the table could not be calculated as an appropriate verification threshold criterion could not be identified; ^c^ Could not calculate the missing frequencies in the table as the authors used a verification threshold criterion based on the predicted versus measured $\dot{V}O_{2\max}$ in the CPET and verification phase; ^d^ Total study sample was 135 participants of which 114 performed both a CPET and verification phase. CSI = continuous step-incremented; DSI = discontinuous step-incremented.

## Discussion

### Main findings

To the best of our knowledge, this is the first systematic review and meta-analysis to investigate the utility of the verification phase for confirming $\dot{V}O_{2\max}$ in special and clinical groups. The major findings were: a) overall, the highest $\dot{V}O_{2}$ attained in the CPET was not statistically significant different to that obtained in the verification phase across all primary studies included; b) subgroup analysis showed there were no statistically significant differences in the highest $\dot{V}O_{2}$ attained in the CPET and verification phase for specific groups; c) across all studies, the difference between the highest $\dot{V}O_{2}$ attained in the CPET and verification phase was not affected by test protocol characteristics; d) participant-level verification phase data might be useful for providing evidence of whether the CPET likely elicited $\dot{V}O_{2\max}$ in a given person; e) a $\dot{V}O_{2}$ plateau in the CPET does not always confirm $\dot{V}O_{2\max}$ since the verification phase $\dot{V}O_{2}$ is sometimes higher in the presence of a $\dot{V}O_{2}$ plateau; and f) the included studies did not report any adverse events associated with the verification phase.

The mean absolute difference in the highest $\dot{V}O_{2}$ attained in the verification phase and CPET was less than 1% for the 30 studies included in the meta-analysis. This difference was similar to that found in our recent meta-analysis that investigated the utility of the verification phase in apparently healthy adults [29]. The global analysis (see Fig 3) revealed a statistically significantly higher mean $\dot{V}O_{2}$ in the CPET for only one study [44]. This study involved 14 elite para ice hockey players and was the only study that used a ski ergometer [44] involving an upper-body poling technique. The CPET involved an initial WR associated with a value of 11 on the Borg RPE scale, followed by WR increments of 10 W every 30 s. The verification phase was performed at 110% of WR_max_ achieved in the CPET. The rapid WR increments during the CPET led to a relatively short mean ± SD test duration of 365 ± 58 s (unpublished data) and a higher peak WR than would have been the case for a longer protocol. Consequently, the verification phase WR might have been excessively high and resulted in a mean ± SD test duration of only 90 ± 13 s (unpublished data), likely due to early local fatigue of the upper-body musculature. These findings indicate that a longer CPET duration and/or lower WR for the verification phase are required for achieving a verification phase of sufficient length in athletes with lower-limb impairments performing upper-body exercise modalities.

A plausible explanation for the contrasting findings between this study and the other 29 meta-analysed studies is that the verification phase workload was presumably too high to attain similar $\dot{V}O_{2\max}$ values because it induced fatigue of the limited active upper body musculature. These results suggest the adoption of a lower percentage of peak work rate might be a better strategy for this specific population.

### Paediatric group

Similar $\dot{V}O_{2\max}$ values were observed between the CPET and verification phase in paediatric participants. Accordingly, two original studies found no significant difference in the $\dot{V}O_{2\max}$ attained in the CPET and verification phase in children [30, 55]. Conversely, a study involving 128 children and adolescents (76 male and 52 females; aged 9.3–17.4 years with and without overweight [71]), reported a significantly higher $\dot{V}O_{2\max}$ in the CPET, although the authors reported a strong correlation (r = 0.94) between the tests. When comparing the highest $\dot{V}O_{2}$ values obtained in the CPET and verification phase on an participant-level basis, 88% (n = 112) of children and adolescents had their highest $\dot{V}O_{2}$ in the CPET verified as their ‘true’ $\dot{V}O_{2\max}$ (< 5% difference between protocols). For the remaining 12% of participants who did not have their $\dot{V}O_{2\max}$ verified, the highest $\dot{V}O_{2}$ recorded in the verification phase was 6–23% higher than in the CPET. The authors recommended the verification phase be used to confirm $\dot{V}O_{2\max}$ attainment in paediatric individuals. In another study [46], eight non-obese children had their $\dot{V}O_{2\max}$ confirmed in the verification phase (< 3% difference in $\dot{V}O_{2\max}$), while 15 achieved a higher $\dot{V}O_{2}$ (> 3% difference in $\dot{V}O_{2\max}$ between protocols) in the verification phase. Consequently, these data suggest the suitability of the verification phase to confirm $\dot{V}O_{2\max}$ has been attained in non-obese, overweight, and obese children.

### Wheelchair group

The subgroup of people using a wheelchair attained similar highest $\dot{V}O_{2}$ values between protocols. Of the three studies included in this sub-analysis, two investigated the verification phase as the focus of the study [42, 56]. The first recruited 10 individuals with spinal cord injury, who performed a verification phase at 105% of the peak work rate (WR_peak_) attained in the CPET, attained highest $\dot{V}O_{2}$ values that were not significantly different between the CPET and verification phase. Hence, the authors recommended a verification phase to confirm ‘true’ $\dot{V}O_{2\max}$ in people with spinal cord injury. Similarly, 24 trained wheelchair athletes (tetraplegics, paraplegics and non-spinal cord-injured) had their $\dot{V}O_{2\max}$ confirmed through a verification phase performed at the same treadmill speed, but a gradient of 0.3% to 0.6% higher than that of the CPET treadmill gradient.

### Chronic respiratory group

The verification phase also confirmed $\dot{V}O_{2\max}$ in the chronic respiratory group. Fourteen paediatric and 28 adults with cystic fibrosis performed a supra peak verification phase at 110% WR_max_. Twelve paediatric (85.7%) and 27 adults (96.4%) had their $\dot{V}O_{2\max}$ confirmed through the verification phase by applying a threshold of 9% difference between CPET and verification phase protocols, which represented the within-subject variability in $\dot{V}O_{2\max}$ for this population [50]. No exercise-induced hypoxemia or perceived discomfort were reported. The authors concluded that a verification phase should be incorporated to confirm that a $\dot{V}O_{2\max}$ has been attained. Sixteen adolescents with mild-to-moderate cystic fibrosis (8 males and 8 females; age 14.6 ± 1.7 years) also achieved similar highest $\dot{V}O_{2}$ values in the CPET and verification phase [78]. Notably, the verification phase was reported as being well tolerated. These results reinforce the robustness of the verification phase as a tool to confirm $\dot{V}O_{2\max}$ in people with chronic respiratory limitations.

### Geriatric group

Regarding the geriatric group, our results demonstrated no significance difference in the highest $\dot{V}O_{2}$ observed in the CPET and verification phase. The lack of difference in $\dot{V}O_{2\max}$ between protocols was reported in eight older adults who performed a verification phase at 85% WR_max_ and 23 older adults who exercised at 105% WR_max_. Moreover, the highest $\dot{V}O_{2}$ values observed in the CPET and verification phase were highly correlated (r = 0.99). The authors emphasised that only minor within-individual differences were observed between the highest $\dot{V}O_{2}$ elicited during the CPET and verification phase [65]. Another study investigated the applicability of the verification phase performed at 85% (n = 22) and 110% (n = 20) WR_max_ in older adults [77]. The mean highest $\dot{V}O_{2}$ across participants was not significantly different between both sub and supra peak verification phases and the associated CPET. Although not included in any of our subgroup analyses, Bowen, Cannon [49] showed that group highest $\dot{V}O_{2}$ values were similar between CPET and a verification protocol performed at 95% WR_peak_ (14.5 ± 3.0 vs. 14.7 ± 3.1 mL·kg^−1^·min^−1^) within 24 older adults with chronic heart failure. However, within-subject comparisons confirmed $\dot{V}O_{2\max}$ in only 14 of 24 patients. Importantly, the verification phase protocol was cited to accurately and reliably confirm (or refute) $\dot{V}O_{2\max}$ along with a measurement sensitivity (95% confidence interval) that is specific for each individual. Likewise, using a similar protocol, Manresa-Rocamora, Fuertes-Kenneally [58] showed $\dot{V}O_{2\max}$ was confirmed in 11 (52.4%) patients and not confirmed in 10 (47.6%). The authors concluded that a submaximal verification phase performed at 95% WR_peak_ is a safe and suitable method to determine $\dot{V}O_{2\max}$ in patients with heart failure with reduced ejection fraction. However, this primary study was not included in the meta-analysis since the authors reported non-parametric variables related to $\dot{V}O_{2}$ and verification phases, and a non-parametric 95% CI for the differences were used to compare the CPET and verification phase.

### Metabolic group

Our final group sub-analysis within the metabolic group revealed a trend for an attenuated $\dot{V}O_{2}$ in the CPET compared to the verification phase in individuals with overweight or obesity, with or without metabolic disease or prehypertension. Notably, all six studies in the subgroup analysis used cycle ergometry. In a trial with four verification phases performed at 80, 90, 100 and 105% WR_max_, eight of nine men with obesity attained a higher $\dot{V}O_{2}$ in the verification phase [57]. Although not statistically significant, the highest $\dot{V}O_{2}$ during the verification phase at 90% WR_max_ was 0.24 L·min^-1^ higher than during the CPET, which is a 7% difference. Other data from studies with large samples of individuals who are sedentary, have obesity, and metabolic disease, indicate that the magnitude (3%-9%) and prevalence (40% of people with overweight/obesity) of the underestimation of $\dot{V}O_{2\max}$ during a CPET is high. Regardless, the results from the current meta-analysis and these primary studies indicate that the verification phase appears to be a robust method for confirming $\dot{V}O_{2\max}$ in individuals with overweight or obesity, with or without metabolic disease or prehypertension.

### Verification phase characteristics

Regarding verification phase characteristics, Fig 5 illustrates the combined effects of work rate, recovery mode, use of a verification phase threshold, day of the test, and protocol duration. No significant results were found for the combined analysis, nor when each category was analysed separately. Consequently, a specific verification phase protocol cannot currently be recommended for any of the groups included in the present systematic review. These results agree with those found in apparently healthy adults, where the verification phase was not affected by test protocol characteristics [29]. Although $\dot{V}O_{2\max}$ was similar among different protocols, notably, no included study applied a sub WR_peak_ verification phase on a treadmill. It therefore remains unclear if relative exercise work rate affects the suitability of the verification phase for confirming $\dot{V}O_{2\max}$ on a treadmill in special and clinical groups.

Regarding the mode of recovery (i.e., active or passive) between the CPET and verification phase, our meta-analysis demonstrated that the 20 mL·min^-1^ mean difference between the highest $\dot{V}O_{2}$ values observed in the CPET and verification phase was not statistically significant. Similar to a recent systematic review that investigated the utility of the verification phase in apparently healthy adults [29], we did not find any study that compared active and passive recoveries. In the present systematic review, the time between the CPET and verification phase ranged from 4 to 25 minutes in studies where these tests were performed on the same day. Although no single study compared the verification phase conducted on the same day versus a different day to the CPET, no statistically significant difference was found in the present review after combining the results. We therefore recommend either an active or passive recovery after the CPET, with no need for an additional visit on a separate day.

Considering the importance of a verification phase being individually analysed to identify those who confirmed their $\dot{V}O_{2\max}$, we suggest the adoption of a threshold criterion to compare the differences between the CPET and verification phase. The threshold criterion value has commonly been based on the reproducibility of $\dot{V}O_{2\max}$ during the CPET and specific to the metabolic cart that was used, or an arbitrary 2–3% difference has been used. Alternatively, some studies have calculated the differences between measured and predicted $\dot{V}O_{2\max}$ [45–47, 61, 62]. However, we did not obtain significant differences between studies that did or did not apply a threshold criterion. We systematically recorded whether authors of primary studies commented specifically on participant level data. In fact, 22 studies (51%) in the present systematic review discussed participant level differences between the highest $\dot{V}O_{2}$ attained in the CPET and verification phase. We strongly suggest future researchers report participant-level data, since the absence of any significant mean differences in the highest $\dot{V}O_{2}$ attained in the CPET and verification phase, may mask individuals who attain a practically significant higher $\dot{V}O_{2}$ in the verification phase.

The combined effects of verification phase duration (i.e., short < 80 s, medium 81–120 s, and long > 120 s) resulted in no statistically significant difference between the highest $\dot{V}O_{2}$ observed in the CPET and verification phase. Similarly, a previous study did not find any significant correlations between different durations at 110% WR_max_ for the highest $\dot{V}O_{2}$ attained in the CPET and verification phase [77]. Furthermore, 12 paediatric participants with cystic fibrosis confirmed their $\dot{V}O_{2\max}$ and two showed 9% higher $\dot{V}O_{2\max}$ values during a verification phase performed at 110% WR_max_ (76 ± 22 s). A brief duration may be inadequate to allow sufficient time for oxygen uptake to achieve maximal values, especially in individuals having slow O_2_ kinetics such as those with metabolic or respiratory disease [81–83]. Verification phases shorter than 80 s might elicit $\dot{V}O_{2\max}$, however, we recommend future studies implement strategies to avoid inappropriately short verification phases and early termination, such as the adoption of multistage verification protocols that incorporate submaximal and supra peak (in relation to the CPET WR_max_) work rates.

### Participant-level data

Whether the verification phase confirmed that $\dot{V}O_{2\max}$ was likely elicited during the CPET was highly variable across the 23 studies that either reported this information directly, or where participant-level data were available to obtain this information. For example, the verification phase elicited a higher $\dot{V}O_{2}$ than the CPET in 0% to 88.9% of participants across studies. This large variability between studies is likely due to differences in the CPET and verification phase protocols that were used for the various special and clinical groups involved.

The verification phase failed to confirm the highest $\dot{V}O_{2}$ in all participants in one study involving para ice hockey players [44], which included a CPET with rapidly-incrementing work rates and a verification phase performed at 110% of the WR_max_ achieved in the CPET. A longer CPET duration with a slower ramp rate and/or a lower WR in the verification phase might be more effective in eliciting a higher $\dot{V}O_{2}$ when using upper-body exercise modalities in athletes with lower-limb impairments. In other studies where participants elicited a higher $\dot{V}O_{2}$ in the CPET, the percentage ranged from 3.6% to 35%. The study that observed 35% involved 22 apparently healthy older adults that performed verification phases at 85% and 110% WR_max_ [77]. The authors concluded that 85% WR_max_ was preferable for this population as it more likely confirmed $\dot{V}O_{2\max}$.

Four out of seven people after stroke achieved a $\dot{V}O_{2}$ that was > 3% higher in the verification phase versus the CPET (range 4.7% to 24.1%) [60]. Similar to the para ice hockey athletes [44], it is unclear whether the same limiting factors during the CPET also occurred during the verification phase, leading to submaximal $\dot{V}O_{2}$ values in both protocols. Twelve of 18 (66.7%) children elicited a higher $\dot{V}O_{2}$ in the verification phase compared to the CPET [45]. A notable finding is five of the seven children that exhibited a $\dot{V}O_{2}$ plateau during the CPET elicited a higher $\dot{V}O_{2}$ during the verification phase. One study involving nine men with obesity compared verification phases performed at 80%, 90%, 100% and 105% of WR_max_ achieved in the CPET [57]. Eight of the men attained a higher $\dot{V}O_{2}$ during a verification phase, with the submaximal verification phase performed at 90% WR_max_ eliciting the highest $\dot{V}O_{2}$ values. These findings are similar to those observed in older adults in that a sub-peak verification phase during testing appears preferable for adults with obesity, at least during cycle ergometry. In another study including adults with obesity [72], a verification phase performed at 100% WR_max_ elicited a $\dot{V}O_{2}$ that was 2% to 21% higher than in the CPET in 13 out of 19 participants. A plausible explanation for these findings is that the CPET work rate increment of 30 W·min^-1^ for men was inappropriately high for this group, resulting in submaximal $\dot{V}O_{2}$ values during the CPET. Two studies included older adults with hypertension [32, 74]. In the first study [32], 18 out of 33 participants achieved a higher $\dot{V}O_{2}$ in the verification phase compared to the value obtained in the CPET (ranging from 3 to 22.1%). Notably, 29 out of 33 tests would have been validated as eliciting $\dot{V}O_{2\max}$ if the traditional criteria were utilised (i.e. false positives). In the second study [74], 9 out of 12 participants obtained a verification-derived $\dot{V}O_{2}$ that was 4.9 to 21% higher than the value observed in the CPET at baseline and after a 12 week exercise program. All participants but one would have had their tests validated as eliciting $\dot{V}O_{2\max}$ if traditional criteria were applied. Results from the present systematic review therefore question the validity of the $\dot{V}O_{2}$ plateau as the primary criterion for establishing the occurrence of $\dot{V}O_{2\max}$ [13] and highlights the importance of performing a verification phase even in the presence of a $\dot{V}O_{2}$ plateau in the CPET.

Midgley et al. [12] suggested that it is unlikely that a person would give identical submaximal efforts in the CPET and verification phase, thereby rationalising that similar highest $\dot{V}O_{2}$ values in the CPET and verification phase likely verifies that $\dot{V}O_{2\max}$ has been elicited. Whether this supposition is true is debatable, especially in certain clinical populations where exercise might be terminated due to symptoms related to the clinical condition. Examples are people with pulmonary disease where undue breathlessness typically results in test termination before any cardiovascular limitation [84], severely deconditioned individuals with very poor exercise tolerance, and those conditions associated with chronic pain made worse with exercise. In such people, the verification phase might simply verify the reproducibility of the $\dot{V}O_{2}$ associated with symptom tolerance (i.e., $\dot{V}O_{2peak}$). This is likely also true for CPET involving upper body ergometry, where the CPET is often limited by peripheral fatigue [85].

### Limitations and recommendations for future research

A limitation of the present systematic review is that 13 (30%) of the 43 studies included in the systematic review were not included in the meta-analysis due to unsuccessful attempts to acquire the required unpublished information from the corresponding authors for these studies. Some authors responded to our correspondence declaring they no longer have access to the requested data. In regards group-level data, the verification phase appears useful for helping verify that, on average, a specific CPET protocol elicited $\dot{V}O_{2\max}$ in special and clinical groups. A verification phase therefore seems unnecessary for this purpose if a new study uses the same CPET protocol with the same population as a previous study that confirmed the CPET was appropriate for eliciting $\dot{V}O_{2\max}$ using a verification phase.

Another limitation of the present systematic review is that participant-level data were available for only seven of the 43 reviewed studies, limiting inferences that could be made. We recommend that participant-level data are made available for future studies. Further research is needed to optimise verification phase protocols so that evidence-based guidelines can be published in the future. This includes identifying robust verification threshold criteria to establish when the highest $\dot{V}O_{2}$ values elicited in the CPET and verification phases are sufficiently similar for $\dot{V}O_{2\max}$ or $\dot{V}O_{2peak}$ to be ‘verified’. Few studies have directly compared test protocols and this is a particularly important area for future research.

Further critical discussion is also needed among researchers regarding how the verification phase should be interpreted when applied to participant-level data. For example, In the first edition of the Canadian Association of Sports Sciences guidelines for the physiological testing of high-performance athletes, Thoden et al. [86] suggested that the highest $\dot{V}O_{2}$ value elicited in either the CPET or verification phase should be regarded as $\dot{V}O_{2\max}$. In the second edition of the guidelines, however, Thoden et al. [87] recommended that an increase in the highest $\dot{V}O_{2}$ elicited in the verification phase that is not more than 2% higher than that elicited in the CPET, verifies $\dot{V}O_{2\max}$ was elicited in the CPET. At the participant level, the verification phase can therefore be used to verify whether $\dot{V}O_{2\max}$ was likely elicited in the CPET or used simply as another opportunity for a person to elicit $\dot{V}O_{2\max}$.

## Conclusion

The main finding was that the mean difference in $\dot{V}O_{2}$ between the CPET and associated verification phase was similar. In other words, the verification phase confirmed that $\dot{V}O_{2\max}$ had been attained in the CPET. Moreover, a 10-to-15-minute recovery phase and short verification phase following a CPET appears to be safe, well-tolerated, and time-efficient for a diverse range of special and clinical groups. Unlike traditional $\dot{V}O_{2\max}$ criteria, the application of the verification phase was not affected by differences in test protocol or procedures, or participant characteristics, except perhaps for those with overweight or obesity. In those with overweight or obesity, the $\dot{V}O_{2}$ attained in the CPET was significantly lower than that obtained in the verification phase. For individuals with obesity and apparently healthy older adults, the selection of sub peak work rates above critical power is desirable. For the remaining groups, it remains somewhat unclear which work rate is most suitable. For paediatrics, a verification phase of 100–105% WR_max_ conducted 15-min after the CPET on a cycle or treadmill has been useful. For the wheelchair group, we can only advise to use a verification phase < 110% WR_max_. Regarding individuals with chronic respiratory problems, a cycle ergometer is applicable for a verification phase of 3-min at 20 W, then 110% WR_max_, performed after 5-min of active rest and 10-min of passive rest after the CPET. Older adults, including those with chronic heart failure, may perform a verification phase 5–10 min after the CPET, either on a cycle ergometer or treadmill. If cycling is chosen, a work rate equal to 85–95% WR_peak_ would optimise $\dot{V}O_{2\max}$ attainment. On a treadmill, 2-min at 50%, 1-min at 70%, and then one stage higher than WR_peak_ is recommended. Lastly, adults with obesity or metabolic conditions may perform a verification phase above critical power on a cycle ergometer up to 110% WR_peak_ on the CPET.

Some researchers might decide not to conduct a verification phase where results from the present meta-analysis indicate that particular CPET test protocols and procedures for certain special and clinical groups appear to elicit ‘true’ $\dot{V}O_{2\max}$ values. However, there remains the issue of identifying whether a participant has likely elicited $\dot{V}O_{2\max}$ during a CPET or elicited a submaximal $\dot{V}O_{2}$ value due to early test termination. Considering the limitations associated with traditional $\dot{V}O_{2\max}$ criteria, the verification phase remains a reasonable time-efficient alternative in special and clinical populations; however, further research and critical debate are required before robust evidence-based guidelines can be provided.

## Supporting information

S1 Checklist PRISMA 20009 checklist. (DOCX)

S1 Fig Search strategies for all databases. (DOCX)

S2 Fig Modified downs and Black checklist. (DOCX)
