# Supplementary material for: Confirming the attainment of maximal oxygen uptake within special and clinical groups: A systematic review and meta-analysis of cardiopulmonary exercise test and verification phase protocols
Source: PLoS One. 2024 Mar 28;19(3):e0299563. doi: 10.1371/journal.pone.0299563 (PMC10977812; doi:10.1371/journal.pone.0299563)
Supplement: S1 Fig — (DOCX) [file pone.0299563.s002.docx]

**Supplementary appendix: Search strategies for all databases**

1. Search Strategy for MEDLINE (Pubmed):

(("Exercise Test"[mh] OR "Exercise Test" OR "Exercise Testing" OR "Treadmill Test" OR "Treadmill Testing" OR "Bicycle Test" OR "Bicycle Testing" OR "Cardiopulmonary Exercise Test" OR "Cardiopulmonary Exercise Testing" OR "CPET" OR "Incremental Test" OR "Incremental Testing" OR "Incremental Exercise" OR "Graded-Exercise Test" OR "Graded-Exercise Testing" OR "GXT" OR "Step-incremented" OR "Step-exercise" OR "Ramp Test" OR "Ramp Testing" OR "Ramp-incremental") AND ("Verification Phase" OR "Verification Test” OR "Verification Testing" OR "Verification Bout" OR "Verification Stage" OR "Verification Criteria" OR "Supramaximal Test" OR "Square-wave" OR "Exhaustive Test" OR "Exhaustive Testing" OR "CWR" OR "RISE")) AND ("Oxygen Consumption"[mh] OR "Oxygen Consumption" OR "Oxygen Uptake" OR "VO2" OR "VO2 max" OR "VO2max" OR "VO2 peak" OR "VO2peak") Sort by: Most Recent

2. Search Strategy for Web of Science:

TÓPICO: (“Exercise Test”  OR “Exercise Testing”  OR “Treadmill Test”  OR “Treadmill Testing”  OR “Bicycle Test”  OR “Bicycle Testing”  OR “Cardiopulmonary Exercise Test”  OR “Cardiopulmonary Exercise Testing”  OR “CPET”  OR “Incremental Test”  OR “Incremental Testing”  OR “Incremental Exercise”  OR “Graded-Exercise Test”  OR “Graded-Exercise Testing”  OR “GXT”  OR “Step-incremented”  OR “Step-exercise”  OR “Ramp Test”  OR “Ramp Testing”  OR “Ramp-incremental”) AND TÓPICO: (“Verification Phase”  OR “Verification Test”  OR “Verification Testing”  OR “Verification Bout”  OR “Verification Stage”  OR “Verification Criteria”  OR “Supramaximal Test”  OR “Square-wave”  OR “Exhaustive Test”  OR “Exhaustive Testing”  OR “CWR”  OR “RISE”) AND TÓPICO: (“Oxygen Consumption”  OR “Oxygen Uptake”  OR “VO2”  OR “VO2 max”  OR “VO2max” OR “VO2 peak”  OR “VO2peak”)

Tempo estipulado: Todos os anos. Índices: SCI-EXPANDED, SSCI, A&HCI, CPCI-S, CPCI-SSH, ESCI.

3. Search Strategy for SCOPUS:

“Exercise Test” OR “Exercise Testing” OR “Treadmill Test” OR “Treadmill Testing” OR “Bicycle Test” OR “Bicycle Testing” OR “Cardiopulmonary Exercise Test” OR “Cardiopulmonary Exercise Testing” OR “CPET” OR “Incremental Test” OR “Incremental Testing” OR “Incremental Exercise” OR “Graded-Exercise Test” OR “Graded-Exercise Testing” OR “GXT” OR “Step-incremented” OR “Step-exercise” OR “Ramp Test” OR “Ramp Testing” OR “Ramp-incremental” AND “Verification Phase” OR “Verification Test” OR “Verification Testing” OR “Verification Bout” OR “Verification Stage” OR “Verification Criteria” OR “Supramaximal Test” OR “Square-wave” OR “Exhaustive Test” OR “Exhaustive Testing” OR “CWR” OR “RISE” AND “Oxygen Consumption” OR “Oxygen Uptake” OR “VO2” OR “VO2 max” OR “VO2max” OR “VO2 peak” OR “VO2peak” AND NOT INDEX (medline)

4. Search Strategy for SPORTDiscus (Ebsco):

( “Exercise Test” OR “Exercise Testing” OR “Treadmill Test” OR “Treadmill Testing” OR “Bicycle Test” OR “Bicycle Testing” OR “Cardiopulmonary Exercise Test” OR “Cardiopulmonary Exercise Testing” OR “CPET” OR “Incremental Test” OR “Incremental Testing” OR “Incremental Exercise” OR “Graded-Exercise Test” OR “Graded-Exercise Testing” OR “GXT” OR “Step-incremented” OR “Step-exercise” OR “Ramp Test” OR “Ramp Testing” OR “Ramp-incremental” ) AND ( “Verification Phase” OR “Verification Test” OR “Verification Testing” OR “Verification Bout” OR “Verification Stage” OR “Verification Criteria” OR “Supramaximal Test” OR “Square-wave” OR “Exhaustive Test” OR “Exhaustive Testing” OR “CWR” OR “RISE” ) AND ( “Oxygen Consumption” OR “Oxygen Uptake” OR “VO2” OR “VO2 max” OR “VO2max” OR “VO2 peak” OR “VO2peak” )

5. Search Strategy for EMBASE

('exercise test' OR 'exercise testing' OR 'treadmill test' OR 'treadmill testing' OR 'bicycle test' OR 'bicycle testing' OR 'cardiopulmonary exercise test' OR 'cardiopulmonary exercise testing' OR 'cpet' OR 'incremental test' OR 'incremental testing' OR 'incremental exercise' OR 'graded-exercise test' OR 'graded-exercise testing' OR 'gxt' OR 'step-incremented' OR 'step-exercise' OR 'ramp test' OR 'ramp testing' OR 'ramp-incremental') AND ('verification phase' OR 'verification test' OR 'verification testing' OR 'verification bout' OR 'verification stage' OR 'verification criteria' OR 'supramaximal test' OR 'square-wave' OR 'exhaustive test' OR 'exhaustive testing' OR 'cwr' OR 'rise') AND ('oxygen consumption' OR 'oxygen uptake' OR 'vo2' OR 'vo2 max' OR 'vo2max' OR 'vo2 peak' OR 'vo2peak')

**Confirming the attainment of maximal oxygen uptake within special and clinical groups: a systematic review and meta-analysis of cardiopulmonary exercise test and verification phase protocols**

Victor A. B. Costa^1,2^ (ORCID: 0000-0002-0804-656X), Adrian W. Midgley^3^* (ORCID: 0000-0002-6139-4168), Julia K. Baumgart^4^ (ORCID: 0000-0001-5628-6050), Sean Carroll^5^, Todd A. Astorino^6^ (ORCID: 0000-0003-0492-0173), Gustavo Z. Schaun^7^ (ORCID: 0000-0003-3339-714X), Guilherme F. Fonseca^1,2^ (ORCID: 0000-0003-0635-4427), and Felipe A. Cunha^1,2^ (ORCID: 0000-0002-8912-5656)

1) Graduate Program in Exercise Science and Sports, University of Rio de Janeiro State, Rio de Janeiro, Brazil.

2) Laboratory of Physical Activity and Health Promotion, University of Rio de Janeiro State, Rio de Janeiro, Brazil.

3) Department of Sport and Physical Activity, Edge Hill University, Ormskirk, England.

4) Centre for Elite Sports Research, Department of Neuromedicine and Movement Science, Norway, University of Science and Technology, Trondheim, Norway.

5) School of Sport, Exercise and Rehabilitation Sciences, University of Hull, Hull, England.

6) Department of Kinesiology, California State University, San Marcos, CA, USA.

7) Centre for Sport Science and University Sports, University of Vienna, Vienna, Austria.

*** Corresponding author**:

Prof Adrian Midgley. Department of Sport and Physical Activity, Edge Hill University. Ormskirk, L39 4QP, England. E-mail: [Midglead@edgehill.ac.uk](mailto:Midglead@edgehill.ac.uk)

Journal name: Sports Medicine
