# Supplementary material for: Confirming the attainment of maximal oxygen uptake within special and clinical groups: A systematic review and meta-analysis of cardiopulmonary exercise test and verification phase protocols
Source: PLoS One. 2024 Mar 28;19(3):e0299563. doi: 10.1371/journal.pone.0299563 (PMC10977812; doi:10.1371/journal.pone.0299563)
Supplement: S2 Fig — (DOCX) [file pone.0299563.s003.docx]

**Modified Downs and Black checklist**

**Color coding**

Black = original questions

Blue = original comments

~~Blue~~ = we deleted parts of the original comments

Green = specifications/adjustments made for this review

**Note that the following words have been changed from the original format**:

“Patient” was replaced with “participant”

“Principal confounders” was replaced with “participant characteristics”

|  | Yes | No | U | N/A | Comments |
| --- | --- | --- | --- | --- | --- |
| *Reporting* | | | | | |
| *1. Is the hypothesis/aim/objective of the study clearly described?* This item is only rated as a Yes, if the aim OR purpose OR hypothesis are clearly described. The aim OR purpose OR hypothesis do not necessarily need to include the cardiopulmonary exercise test (CPET) to exhaustion and/or verification phase, as we acknowledge that this may not be the main aim OR purpose of the included studies. |  |  |  |  |  |
| *2. Are the main outcomes to be measured clearly described in the introduction or methods section?* If the main outcomes are first mentioned in the results section, the question should be answered with a no. |  |  |  |  |  |
| *3. Are the characteristics of the participants included in the study clearly described?* In cohort studies and trials, inclusion and/or exclusion criteria should be given. In case-control studies, a case-definition and the source for controls should be given. It needs to be clear that all study participants belong to one of the special populations targeted in this review (i.e., children, older adults, people with a disability, people with obesity). If it this is clear and it is clear from the inclusion criteria which participants are excluded, rate this item as a Yes. If this is not clear and the study does not mention any exclusion criteria, rate this item as a No. |  |  |  |  |  |
| *4. Are the interventions of interest clearly described?* Treatments and placebo (where relevant) that are to be compared should be clearly described. For this review the CPET to exhaustion can be considered the control/ placebo and the verification phase the treatment.  For the CPET, the following information needs to be provided:   - Duration of stages and magnitude of increments - Duration of the test   For the verification phase, the following information needs to be provided:   - If the test was sub- or supra-peak or at the peak power output/speed obtained during the maximal test to exhaustion including the work rate/ speed at which it was performed - Duration of the test   Furthermore, the following information needs to be included:   - Exercise modality used for both tests - Time and type of recovery between tests (if performed on separate tests, it needs to be specified that the testing was performed at the approximately same time of day)     Both tests need to be described in sufficient detail for this item to be ranked as a yes (2 points) or yes (1 point). **Two points** are given if **all six items** are described. **One point** is given if **four-five** of the six items are described. |  |  |  |  |  |
| *5. Are the participant characteristics ~~distribution of principal confounders in each group of subjects to be compared~~ clearly described?* A list of participant characteristics is provided. This contains sex, age, body height and body mass of the participants. For studies on wheelchair users, body height does not need to be provided. In addition, for the clinical populations, sufficient detail needs to be provided in the type of disability/disease. |  |  |  |  |  |
| *6. Are the main findings of the study clearly described?*  Simple outcome data (including denominators and numerators) should be reported for all major findings so that the reader can check the major analyses and conclusions. (This question does not cover statistical tests, which are considered below). In addition, oxygen uptake (VO_2_) and its unit of measurement need to be provided for both the maximal test to exhaustion and the verification phase.  Both (i.e., simple outcome data for the major findings + VO_2_ and its unit of measurement for both tests) need to be sufficiently described for this item to a yes with 2 points. Only providing the ∆% or absolute difference in VO_2_ for both tests is not sufficient. If only one (i.e., simple outcome data for the major findings + VO_2_ and its unit of measurement for both tests) is sufficiently described, it is rated as a yes with 1 point. |  |  |  |  |  |
| *7. Does the study provide estimates of the random variability in the data for the main outcomes?* In non normally distributed data the inter-quartile range of results should be reported. In normally distributed data the standard error, standard deviation or confidence intervals should be reported. ~~If the distribution of data is not described, it must be assumed that the estimates used were appropriate and the question should be answered yes~~. If no mean and SD are calculated and only individual data is provided, rate this item as a No. If it is a case-study this item should be rated as N/A.  In addition, for VO_2_ a measure of central tendency (for example mean or median) and dispersion (for example standard deviation or interquartile range needs to be provided). |  |  |  |  |  |
| *10. Have actual probability values been reported (e.g., 0.035 rather than <0.05) for the main outcomes except where probability is less than 0.001?* This refers to the main outcomes of the study and not necessarily to the comparison of CPET to exhaustion and verification phase. |  |  |  |  |  |
| External validity  All the following criteria attempt to address the representativeness of the findings of the study and whether they may be generalised to the population from which the subjects were derived. | | | | | |
| *11. Were the subjects asked to participate in the study representative of the entire population from which they were recruited?*  The study must identify the source population for participants and describe how the participants were ~~selected~~ recruited. It must be clear what the source population is, and if study participants can be considered representative for this population (e.g., through random sampling). If it is likely that the study participants were selected by convenience sampling, rate this item as a no. ~~Participants would be representative if they comprised the entire source population, an unselected sample of consecutive participants, or a random sample. Random sampling is only feasible where a list of all members of the relevant population exists.~~ ~~Where a study does not report the proportion of the source population from which the patients are derived, the question should be answered unable to determine.~~ |  |  |  |  |  |
| Internal validity – bias | | | | | |
| *17. In trials and cohort studies, do the analyses adjust for different lengths of follow-up of patients, or in case-control studies, is the time period between the intervention and outcome the same for cases and controls?*  Where follow-up was the same for all study patients the answer should be yes. If different lengths of follow-up were adjusted for by, for example, survival analysis the answer should be yes. Studies where differences in follow-up are ignored should be answered no. For this review the maximal test to exhaustion can be considered the control/ placebo and the verification phase the treatment. The time period between the maximal test to exhaustion and the verification phase(s) needs to be specified and standardized within each included study for this item to be rated as a yes. If it is specified that the tests were performed on separate days rate this item as yes. |  |  |  |  |  |
| *18. Were the statistical tests used to assess the main outcomes appropriate?*  The statistical techniques must be appropriate for the data. For example non-parametric methods should be use for small sample sizes. Where little statistical analysis has been undertaken but where there is no evidence of bias, the question should be answered yes. If the distribution of the data (normal or not) is not described it must be assumed that the estimates used were appropriate and the question should be answered yes. At a minimum, the following needs to be described for this item to be ranked as a yes: 1) the specific type of test (only stating that an ANOVA/ T-test or correlations was performed without further specification is insufficient) and 2) what specific outcome measures the test(s) target(s). |  |  |  |  |  |
| *20. Were the main outcome measures used accurate (valid and reliable)?*  For studies where the outcome measures are clearly described, the question should be answered yes. For studies which refer to other work or that demonstrates the outcome measures are accurate, the question should be answered yes. Information on the calibration procedures of the metabolic cart/ ergospirometer needs to be provided. The statement “was calibrated according to the manufacturer’s specifications/instructions” is sufficient. Referring to another study or the manufacturers instructions for the calibration procedures is also sufficient for this item to be ranked as a yes. |  |  |  |  |  |
| *Extra item. Was the sampling method of the ergometer (i.e., mixing chamber or breath-by-breath) and the sampling interval specified?* |  |  |  |  |  |
| Internal validity – confounding (selection bias) | | | | | |
| *21. Were the participants in different intervention groups (trials and cohort studies) or were the cases and controls (case-control studies) recruited from the same population?*  For example, participants for all comparison groups should be selected from the same hospital. The question should be unable to determine for cohort and case-control studies where there is no information concerning the source of patients in the study. The source population in this review is defined as individuals with the same type of special populations targeted in this review (i.e., children, older adults, people with a disability) at a distinct institution/in a distinct area (region, county, country). |  |  |  |  |  |
| Power | | | | | |
| *27. Did the study have sufficient power to detect a clinically important effect where the probability value for a difference due to chance is less than 5%?*  Sample sizes have been calculated to detect a difference from x% and y%. Prospective sample size calculations need to be included in the study for this item to be ranked as a yes. This item may also be ranked as a yes, if a reference is made to another study where sample size calculations were appropriately performed. |  |  |  |  |  |
| Extra item (not included in the quality appraisal score) | | | | | |
| *Extra item. Was the comparison between the incremental test and verification stage a main outcome of the study?* |  |  |  |  |  |

**Confirming the attainment of maximal oxygen uptake within special and clinical groups: a systematic review and meta-analysis of cardiopulmonary exercise test and verification phase protocols**

Victor A. B. Costa^1,2^ (ORCID: 0000-0002-0804-656X), Adrian W. Midgley^3^* (ORCID: 0000-0002-6139-4168), Julia K. Baumgart^4^ (ORCID: 0000-0001-5628-6050), Sean Carroll^5^, Todd A. Astorino^6^ (ORCID: 0000-0003-0492-0173), Gustavo Z. Schaun^7^ (ORCID: 0000-0003-3339-714X), Guilherme F. Fonseca^1,2^ (ORCID: 0000-0003-0635-4427), and Felipe A. Cunha^1,2^ (ORCID: 0000-0002-8912-5656)

1) Graduate Program in Exercise Science and Sports, University of Rio de Janeiro State, Rio de Janeiro, Brazil.

2) Laboratory of Physical Activity and Health Promotion, University of Rio de Janeiro State, Rio de Janeiro, Brazil.

3) Department of Sport and Physical Activity, Edge Hill University, Ormskirk, England.

4) Centre for Elite Sports Research, Department of Neuromedicine and Movement Science, Norway, University of Science and Technology, Trondheim, Norway.

5) School of Sport, Exercise and Rehabilitation Sciences, University of Hull, Hull, England.

6) Department of Kinesiology, California State University, San Marcos, CA, USA.

7) Centre for Sport Science and University Sports, University of Vienna, Vienna, Austria.

*** Corresponding author**:

Prof Adrian Midgley. Department of Sport and Physical Activity, Edge Hill University. Ormskirk, L39 4QP, England. E-mail: [Midglead@edgehill.ac.uk](mailto:Midglead@edgehill.ac.uk)

Journal name: Sports Medicine
